# VALORIS: One-shot and lossless vertical logistic regression for privacy-protecting multi-site health analytics

**DOI:** 10.1038/s41598-026-41936-y

**Published:** 2026-03-08

**Authors:** Félix Camirand Lemyre, Marie-Pier Domingue, Jean-Philippe Morissette, Anita Burgun, Jean-François Ethier

**Affiliations:** 1 Groupe de recherche interdisciplinaire en informatique de la santé (GRIIS) (Mathematics), Université de Sherbrooke, Sherbrooke, Canada; 2 Health Data Research Network Canada, Vancouver, Canada; 3 Institut Imagine, Université Paris Cité, Paris, France; 4 Department of Medical Informatics, Necker Hospital, AP-HP, Paris, France; 5 Groupe de recherche interdisciplinaire en informatique de la santé (GRIIS) (Medicine/Computer science), Université de Sherbrooke, Sherbrooke, Canada

**Keywords:** Computational biology and bioinformatics, Health care, Mathematics and computing, Medical research

## Abstract

Health analytics increasingly relies on variables held by different entities, such as clinical, laboratory, environmental, and genomic data. Due to legal, ethical, and social acceptability constraints, these vertically partitioned data often cannot be shared across organizations holding them. Conducting statistical analyses in such settings requires methods that protect privacy. We introduce VALORIS (Vertically partitioned Analytics under the LOgistic Regression model for Inference in Statistics), a novel method that enables lossless statistical inference (equivalent to the pooled analyses) under a logistic regression model without disclosing any individual-level data–including the outcome variable. VALORIS is a practical, one-shot algorithm that requires no third-party coordinator. The privacy-preserving properties of VALORIS were mathematically assessed, and a privacy-aware setting-dependent framework was provided to ensure individual-data privacy. We demonstrate the accuracy and feasibility of VALORIS through the investigation of potential factors associated with kidney failure among pediatric patients with chronic kidney disease using real health data from Necker-Enfants Malades Hospital. We further validate the proposed algorithm on a larger scale with a reproducible application using the MIMIC-IV database.

## Introduction

Addressing the multidimensional nature of today’s pressing challenges in health sciences increasingly requires statistical analyses that integrate diverse, complementary data types–ranging from clinical, genomic, and laboratory measures to socioeconomic or environmental data. Such data are typically distributed across different organizations, where legal, ethical, social, or commercial constraints often preclude their pooling within a single organization. This fragmentation gives rise to a vertical partitioning, with each data source holding distinct types of information about the same observations. Critically, the constraints often go beyond restricting direct data transfers, and encompass any exchange of information that could enable individual-level data to be retrieved.

Methodologies that enable statistical analyses by coordinating computations across collaborating data-holding organizations, referred to as nodes, without requiring individual-level data to leave their original environments are commonly grouped under the umbrella of distributed statistical analytics. The Health Data Research Network Canada (HDRN), mandated with supporting large-scale collaborative projects involving health data from multiple nodes, has launched a dedicated work stream on distributed analysis to guide the application of distributed procedures in projects seeking this support. However, three criteria must be considered before any procedure can be endorsed.

The first and non-negotiable criterion is that the approach ensures privacy protection–specifically, it entails not only to avoid sharing raw data outside the data nodes, but also to provide formal confidentiality guarantees against reverse-engineering. Alongside privacy, practical feasibility is also essential: the approach should be communication-efficient, recognizing that many data partners operate with limited time and resources. This need for simplicity is particularly important when some partners require human review of all outgoing numerical outputs. A further criterion concerns comparability of results to those obtained in standard centralized settings. The gold standard is to produce results equivalent to those obtained under a centralized (pooled) analysis, meaning that the final outputs of the method can be made arbitrarily close to those of a centralized analysis through a suitable implementation. When this property holds, the method is said to be *lossless*. This comparability criterion is essential to build trust among users accustomed to centralized approaches.

Among existing vertical regression-based approaches that allow statistical inference–parameter estimation and corresponding standard errors, enabling the computation of *p*-values and confidence intervals (CIs), covered from various angles in literature^1–5^, to the best of our knowledge, the only methods that comply with the above-mentioned HDRN criteria are those for the linear model^3,6,7^. By contrast, no method currently achieves this for logistic regression, despite its central role in health analytics. To address this gap, we focus on parameter estimation and standard error computation for standard logistic regression to support HDRN needs and beyond.

To our knowledge, only one method has been proposed for logistic regression in a vertical setting that does not require raw data to leave its original environment^8,9^. Although standard errors, CIs, and hypothesis testing are not explicitly addressed, the procedure can be extended with minor modifications. Yet, as acknowledged by the original authors and highlighted in a subsequent review^10^, the method inherently involves a large number of communication rounds and leaves unresolved concerns about potential data leakage.

As far as we are aware, the only other method addressing these inference tasks and producing results comparable to classical pooled maximum likelihood analyses is the VERTIGO algorithm^11^, together with its extension VERTIGO-CI^12^, applied in recent work^13^. However, this approach requires sharing the outcome variable–local raw data–with every participating node, which is inherently undesirable from a privacy standpoint. Moreover, in prior work, we showed that in many practical settings, running VERTIGO or VERTIGO-CI renders the raw covariate data reconstructible from the disclosed quantities^14^. These findings highlight that the distributed nature of an algorithm does not, on its own, ensure privacy protection, as has also been previously reported^15^, and they underscore the importance of thoroughly assessing the risk that participating parties may reconstruct individual-level data from exchanged quantities in distributed settings.

Taking this into account, the aim of this work is to introduce a novel one-shot, lossless approach for estimating parameters and their standard errors in standard logistic regression with vertically partitioned data. We present the privacy-aware algorithm VALORIS–Vertically partitioned Analytics under the LOgistic Regression model for Inference in Statistics–which avoids sharing any individual-level data, including the response vector, and operates through a single round of communication.

**Fig. 1:**
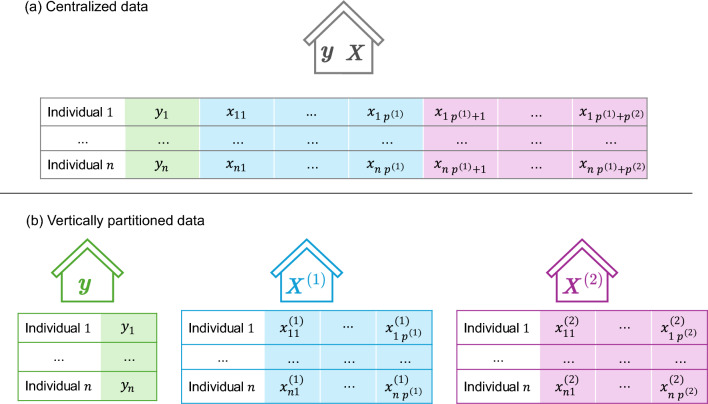
Centralized *vs* Vertically partitioned data settings for the case of two covariate-nodes.

The vertical setting considered for our proposed method is illustrated for the case of three data nodes in Fig. 1. The covariate data matrix ${\boldsymbol{X}} \in \mathbb {R}^{n \times p}$ is partitioned across $K\ge 2$ distinct *covariate-nodes*. Covariate-node *k* holds a matrix ${{\boldsymbol{X}}}^{(k)} \in \mathbb {R}^{n \times p^{(k)}}$ containing the values of its $p^{(k)}$ covariates across all *n* observations, distinct from those stored at other nodes. The response vector ${\boldsymbol{y}}\in \lbrace -1,1\rbrace ^n$ is stored at the *response-node*, and is not shared with the other nodes. Response-node and covariate-node are not mutually exclusive roles, such that a data-node can hold both the response and covariates. We assume throughout that datasets are aligned across partitions ${{\boldsymbol{X}}}^{(1)},\ldots , {{\boldsymbol{X}}}^{(K)}$ and that this alignment corresponds to the structure of ${\boldsymbol{y}}$, i.e., Individual 1 appears in row 1 of all partitions and in the first entry of ${\boldsymbol{y}}$, and so forth.

**Fig. 2:**
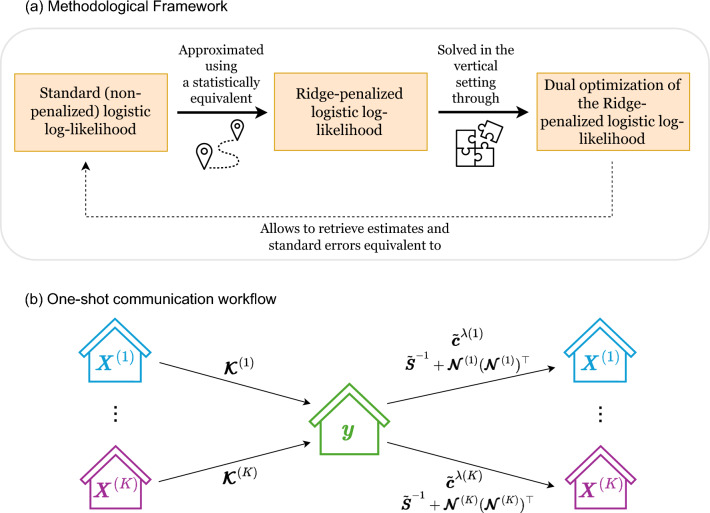
Overview of VALORIS: Vertically partitioned Analytics under the LOgistic Regression model for Inference in Statistics. Details regarding the intermediate numerical outputs exchanged are provided in Methods - *Detailed steps of the VALORIS algorithm*.

Our proposed algorithm VALORIS relies on a single back-and-forth exchange of intermediate numerical outputs between the covariate-nodes and the response-node, with the covariate-nodes disclosing only their local Gram matrices. The lossless property relative to parameter estimation is achieved by leveraging the dual formulation of a ridge-penalized log-likelihood, which, under suitable and commonly satisfied assumptions, provides an approximation to the standard log-likelihood whose approximation error can be made arbitrarily small by selecting a sufficiently small penalty parameter $\lambda$. The lossless property with respect to standard error estimates is achieved through the introduction of an additional parameter $\eta$, which enables the use of a matrix-inversion identity to approximate the target non-penalized standard errors with arbitrarily small approximation error as both $\lambda$ and $\eta$ are taken arbitrarily close to 0. The intermediate numerical outputs sent from the response-node to the covariate-nodes, enabling them to compute the final estimates for their respective variables, were carefully chosen to protect the confidentiality of the response vector by exploiting structural properties of the matrices involved. The methodological approach and communication workflow associated with VALORIS are summarized in Fig. 2.

To demonstrate the feasibility and accuracy of our approach, we present a clinical case study that investigates potential factors associated with kidney failure in pediatric patients with chronic kidney disease (CKD), using de-identified clinical and laboratory structured data from Necker-Enfants Malades Hospital. This analysis extends previous efforts aimed at improving the diagnosis and treatment of renal ciliopathy patients^16^. To validate our method on a larger scale, we provide a reproducible example using the MIMIC-IV database^17^. This application illustrates both the lossless property of the algorithm and its compatibility with larger datasets. To ensure accessibility of our approach, we also made available a third example through a publicly accessible online repository, featuring a synthetic dataset we created along with the accompanying R code for its analysis^18^. To ensure privacy protection, we mathematically assessed the risk that a participating party could reconstruct individual-level data through the proposed procedure by analyzing the equations that could be formed from the quantities available at a given party, where the unknowns correspond to the individual entries of a node’s data matrix, and so while taking into account the nature of the covariates (binary vs continuous).

## Results

### VALORIS to identify characteristics associated with kidney failure among patients at Necker-Enfants Malades Hospital

We first demonstrate the utility and accuracy of VALORIS through the investigation of potential associations between kidney failure within two years of baseline and various characteristics in a cohort of children with CKD. This investigation is part of an initiative to better understand this condition among pediatric patients. To guide our analysis, we drew on a study that proposed two models to predict kidney failure in adults with CKD: a model with four baseline covariates, and a model that included four additional serum measurements^19,20^. The cohort included pediatric patients from Necker-Enfants Malades Hospital (Paris, France) with potential CKD stage 3 or 4 at baseline and complete data ($n = 97$).

**Fig. 3:**
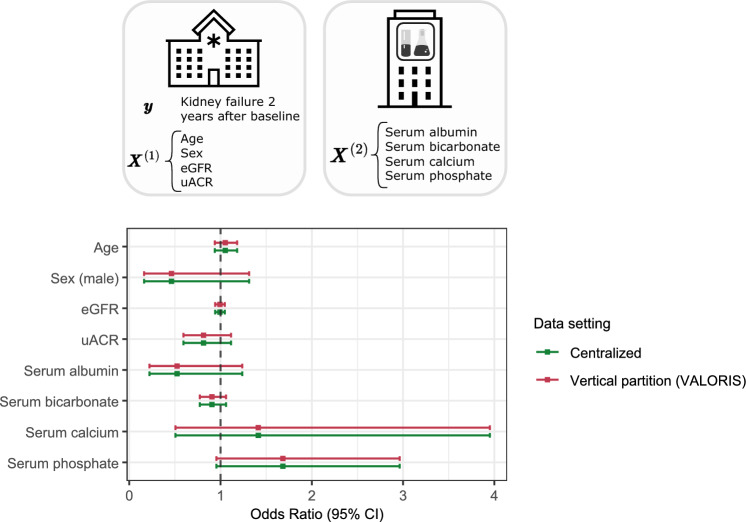
Logistic regression model with kidney failure at two years after baseline as outcome - Vertical partition *vs* Centralized setting.

Baseline covariates and serum measurement are generated by distinct systems, creating a vertical partition across two nodes: one with the binary outcome *kidney failure at two years after baseline* and the covariates *age*, *sex*, *estimated glomerular filtration rate (eGFR)* and *urine albumin-creatinine ratio (uACR)*; the other containing serum measurements. A logistic regression model was assumed for the data and the VALORIS algorithm could therefore be applied. Additional details regarding the parameters setting for VALORIS are provided in Methods - *Detailed steps of the VALORIS algorithm*. To proceed, VALORIS was implemented in R^21^ and implementation is available through the link in Supplementary Notes. The results of the analysis are provided in Fig. 3. They were computed in the vertically partitioned setting using VALORIS, and then compared with those obtained from a non-penalized logistic regression in a centralized setting performed using the *glm* function from the *stats* package in R (version 4.4.1)^21^.

Detailed values for estimates and standards errors are available in Supplementary Table S4. The largest absolute difference observed between VALORIS and the centralized analysis among all parameter estimates and standard errors was $9.41\times 10^{-5}$. The mean absolute difference was $3.03\times 10^{-5}$. It is possible to further reduce this difference by decreasing the values of the parameters $\lambda$ and $\eta$ used in the implementation of the algorithm (see Methods - *Detailed steps of the VALORIS algorithm*), which may require computations to be carried out at higher numerical precision.

### VALORIS to explore associations with death among MIMIC-IV database

Building on the previous section, which established the feasibility and numerical accuracy of VALORIS, we next illustrate its applicability in settings with larger sample sizes and a greater number of covariate-nodes. To this end, we provide a reproducible example using real health data from the MIMIC-IV database^17^, available upon completion of mandatory training, where VALORIS is applied to a larger sample with an increased number of covariate-nodes.

**Fig. 4:**
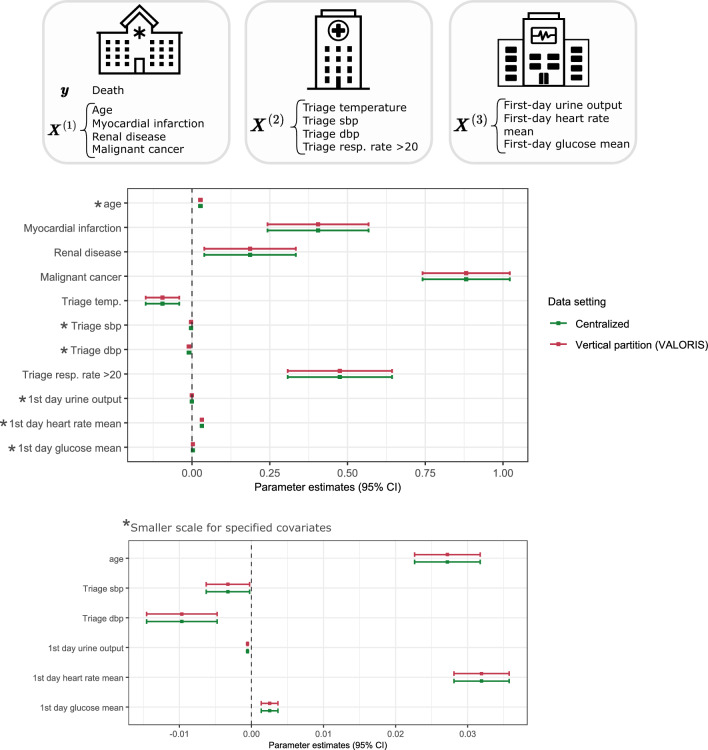
Logistic regression model with MIMIC-IV database and death as outcome - Vertical partition *vs* Centralized setting.

A total of 11 covariates and one outcome partitioned across three modules treated as distinct data nodes were selected. The Hospitalization (HOSP) module was assigned the outcome variable *death* and four covariates. The Emergency Department (ED) and Intensive Care Unit (ICU) modules served as additional covariate-nodes, contributing four and three covariates, respectively. Only complete-case observations were retained, resulting in a sample size of $n = 13677$. The results of the analysis using the VALORIS approach are presented in Fig. 4 and compared with those from the centralized analysis using again the *glm* function from the *stats* package in R (version 4.4.1).

In this application, the mean absolute difference accross all coefficients and standard errors between VALORIS and the centralized setting was $1.65\times 10^{-5}$, with the largest absolute difference being $1.61\times 10^{-4}$. In line with the previous section, these differences could be further reduced by adjusting the parameters $\lambda$ and $\eta$, which may require computations to be carried out at higher numerical precision. The detailed values for all estimates and standard errors are available in Supplementary Table S5.

### Readily executable example of VALORIS - Synthetic data

To offer a readily available application of VALORIS, we generated a synthetic dataset and added it to the GitHub repository^18^. Therein, an example with sample size $n=300$ involving two covariate-nodes respectively holding 3 and 6 covariates can be fully executed, and allows to directly compare the outputs obtained from the application of VALORIS and the centralized analysis with the *glm* function in R.

### Privacy-preserving properties of VALORIS

In the literature, a distributed algorithm is often qualified as *privacy-preserving* if it avoids the direct transmission of individual-level data (see e.g. Duan et al.^22^ stating “The algorithms are privacy-preserving in the sense that patient-level data are not required to be transferred across sites”). However, this level of privacy protection–referred to as Privacy Level I and satisfied by VALORIS–does not suffice to comply with the privacy requirements from organizations such as Necker-Enfants Malades Hospital and HDRN, where, for an algorithm to be considered privacy-preserving, the outgoing information should not allow any participating party to retrieve individual-level data whether it is done directly or using reverse-engineering.

We therefore introduce Privacy Level II, which requires that individual-level data cannot be uniquely recovered from the quantities exchanged or disclosed during the execution of the approach. Specifically, when satisfied, this definition implies that for every entry in the original dataset, there exists at least one *admissible candidate dataset*, consistent with the disclosed quantities, that differs at that entry. Admissible candidate datasets are defined as datasets that (a) are consistent with all exchanged and disclosed quantities available to any party that could, at a given time, attempt to reconstruct the original dataset, and (b) respect the nature of each variable (e.g., binary or continuous).

For assessing compliance with Privacy Level II, we assumed that the algorithm is executed as specified and that it is run in an environment without access to external information (additional scenarios are considered and discussed in detail in Supplementary Methods - *Conservative Scenario*). This corresponds to the *honest-but-curious* adversary setting as defined in the privacy-preserving data mining literature^23^. Our assessment further relied on assumptions that are typically met in practice, including the participation of at least two covariate-nodes–one of which may be co-located with the response-node–in the analysis (see Methods - *Methodology for assessing privacy-preserving properties*).

We first examined the risk that the response-node could reconstruct a covariate-node’s data from its shared Gram matrix, derived from locally centered and scaled data, and found that running VALORIS achieves Privacy Level II regardless of the number or nature of covariates (see Methods - *Methodology for assessing privacy-preserving properties* for details).

Second, verification that the sharing of intermediate quantities from the response-node to the covariate-nodes complies with Privacy Level II is sample-dependent. Under the assumption that at least one continuous covariate is held outside a potential adversarial covariate-node –an assumption automatically satisfied if each covariate-node holds at least one continuous covariate, or if the response-node itself holds covariate data with at least one continuous covariate–, this compliance can be ensured by requiring the response-node to solve a set of feasible-point problems before transmitting any information. This can be addressed using linear programming (see Supplementary Methods - *Privacy-preserving properties - Privacy assessment for the response vector*). An implementation of the resulting verification criterion is available through Supplementary Notes. For all presented applications (Necker-Enfants Malades Hospital data, MIMIC-IV data and simulated data), the empirical criterion was run at the response-node for all covariate-nodes not co-located at the response-node. The criterion was satisfied in all cases.

Third, the overall risk that a party with access to the disclosed parameter estimates, standard errors, or two-tailed *p*-values (after the algorithm has been fully executed) could retrieve a covariate-node’s individual-level data is not specific to VALORIS; it arises in most vertically partitioned analyses when final results are released. Under the assumptions described in Methods - *Methodology for assessing privacy-preserving properties*, including that at least one continuous covariate is held at a covariate-node not co-located with the response-node, we demonstrated that the response-node does not possess sufficient information to uniquely reconstruct the underlying data, and that Privacy Level II is ensured regardless of which estimates or standard errors are disclosed.

## Discussion

Our new algorithm, VALORIS, enables lossless estimation of parameters and their standard errors in logistic regression models with vertically partitioned data. It does not require sharing the response vector across all parties and it explicitly provides transparency on the risk of reconstructing individual-level information through reverse-engineering.

The one-shot communication workflow of VALORIS greatly facilitates the mathematical assessment of its privacy properties, since only a small number of shared quantities need to be examined. This property also makes the method well suited to settings where data nodes must manually review exchanged quantities, and it supports practical deployment regardless of whether an inter-node infrastructure is in place. In contrast, vertically partitioned methods often rely on iterative secure matrix operations or encryption schemes, which impose a substantial communication burden and hinder real-world applicability. VALORIS addresses these limitations by offering a solution that is both practical and feasible.

The efficient communication workflow of VALORIS does not compromise accuracy and statistical equivalence to the centralized setting. The results in Fig. 3 and Fig. 4 show that the estimates and standard errors–or equivalently, the CIs–are nearly identical between the vertical and centralized settings. These findings highlight the accuracy of VALORIS relative to the centralized benchmark. The CKD application serves as a compelling proof of concept for analysis with vertically partitioned data. It motivates conducting further investigation into factors associated with kidney failure in pediatric cohorts, particularly by incorporating additional covariates distributed across different entities. Future distributed analyses that integrate genomic data from national cohorts with clinical data from hospital information systems could help identify renal ciliopathy cases among CKD patients, thereby improving understanding of condition-specific risk factors and supporting targeted interventions.

In many settings, including projects we anticipate undertaking, covariate-nodes will place sufficient trust in the response-node to assume that the algorithm is executed as specified. In real-world collaborations, such expectations can also be formalized in agreements between participating data nodes. In such settings, different sets of numerical results (parameter estimates, standard errors, *p*-values) can be publicly disclosed while ensuring that no reverse-engineering process enables the retrieval of individual-level data. For completeness, we also examined in Supplementary Methods - *Conservative scenario* which results remain disclosable under Privacy Level II in more adversarial settings, across multiple scenarios varying in the nature and number of covariates. Our analysis enables covariate-nodes to make informed decisions about which quantities to disclose, weighing the associated privacy risks against the assumptions they are prepared to accept. This privacy awareness by design distinguishes our method from earlier approaches for statistical analyses under the logistic regression model with vertically partitioned data.

The privacy check underlying Privacy Level II for the response vector plays a central role in the privacy assessment but can incur a non-negligible computational overhead in large-scale settings (see Supplementary Methods - *Privacy-preserving properties - Privacy assessment for the response vector*). While this check is performed only once at the response-node, its runtime increases with the sample size, and depends on the computational environment and solver used, which should be considered when deploying the method in a practical setting. If, however, the privacy criterion fails in its current implementation, meaning that the verification procedure does not identify the admissible candidates required to achieve Privacy Level II, alternative solvers may be used. If the criterion remains inconclusive and such protection of the response individual-level data cannot be formally established, the response-node may decide whether to proceed with the analysis in the absence of Privacy Level II guarantees. As for the predictor-nodes, whether an analysis can proceed in such cases may depend on institutional and regulatory constraints, including data sensitivity and organizational privacy obligations.

Our privacy assessment is intentionally conservative and focuses on establishing sufficient conditions for non-unique reconstruction of individual-level data from the available quantities. When these conditions cannot be verified–such as in settings involving all-binary covariates–this should be interpreted as the absence of a formal guarantee rather than as evidence that reconstruction is uniquely determined; moreover, our analysis does not address computational considerations that may further limit the practical feasibility of reconstruction nor what specific individual-level data may be at risk. Further work could explore privacy assessment strategies tailored to challenging settings such as all-binary covariates, where existing sufficient conditions may be overly conservative.

It remains possible that external information about a dataset could be known or accessible from outside sources. While this lies beyond the scope of the present work, it could be examined in specific applications–for example, in scenarios where the response-node has access to covariate means. In situations where such privacy investigations are judged insufficient, differential privacy has been proposed as an additional layer of protection. However, applying differential privacy may adversely affect the accuracy of analytical results^24^, warranting further work beyond the scope of this paper. The other stream of work is to keep theoretically investigating the level of privacy achieved by distributed methods, in the hope of being able to achieve the objectives without modifying the raw data, nor compromising the results accuracy. It might well be possible, at least for the majority of situations encountered in real health studies.

The work presented in this paper can be extended in several directions, some of which are already under investigation by our team. One important avenue is to move beyond complete-case analyses by incorporating strategies for handling missing data. Another is to adapt the structure of the algorithm to other models in vertical settings.

## Methods

### Background on standard logistic regression

The statistical framework considered in our work is described as follows. Under the logistic regression model, the conditional distribution of $\text {Y}$ given ${\textbf {X}} = {\boldsymbol{x}}\in \mathbb {R}^p$ is given, for $y \in \{-1, 1\}$, as

1 $$\begin{aligned} {{\,\textrm{P}\,}}(\text {Y} = y \mid {\textbf {X}} = {\boldsymbol{x}}) = \frac{1}{1 + \exp \lbrace -y( \beta _{0\star } + {\boldsymbol{x}}^\top {\boldsymbol{\beta }}_{\star })\rbrace }, \end{aligned}$$

where $\beta _{0\star } \in \mathbb {R}$ and ${\boldsymbol{\beta }}_{\star } \in \mathbb {R}^p$ respectively denote the true (but unknown) intercept and covariate parameters (see Supplementary Tables S1, S2 and S3 for detailed notation glossaries). We aim to derive estimates, CIs and/or *p*-values associated with those unknown parameters, using a vertically partitioned sample of *n* independent realizations $\lbrace ({\boldsymbol{x}}_1, y_1), \ldots , ({\boldsymbol{x}}_n, y_n)\rbrace$. When the data are centralized to a single data node, $(\beta _{0\star }, {\boldsymbol{\beta }}_\star )$ of the logistic regression model defined in (1) are typically estimated by solving the following log-likelihood maximization problem:

2 $$\begin{aligned} \max _{\beta _0\in \mathbb {R},{\boldsymbol{\beta }}\in \mathbb {R}^p} \bigg ( \ell _n(\beta _0,{\boldsymbol{\beta }}) := \frac{1}{n}\sum _{i=1}^n \log \left[\frac{1}{1+\exp \lbrace -y_i(\beta _0+ {\boldsymbol{x}}_i^\top {\boldsymbol{\beta }})\rbrace } \right]\bigg ) \,. \end{aligned}$$

The solutions $(\widehat{\beta }_{0}, \widehat{{\boldsymbol{\beta }}})$ of the latter problem, called the maximum likelihood estimates, are generally found using a Newton-Raphson algorithm or a variant of it. The variance-covariance matrix of the maximum likelihood estimates $(\widehat{\beta }_0, \widehat{{\boldsymbol{\beta }}})$ can be estimated by the inverse of the observed Fisher information matrix, defined as

3 $$\begin{aligned} {\boldsymbol{\mathcal {I}}}(\widehat{\beta }_0,\widehat{{\boldsymbol{\beta }}}) : = -\nabla ^2_{\beta _0,{\boldsymbol{\beta }}} \ell _n(\widehat{\beta }_0,{\widehat{\boldsymbol{\beta }}}) = \frac{1}{n}\sum _{i=1}^n \frac{\exp \lbrace y_i(\widehat{\beta }_0+{\boldsymbol{x}}_i^\top \widehat{{\boldsymbol{\beta }}})\rbrace }{[1+\exp \lbrace y_i(\widehat{\beta }_0+{\boldsymbol{x}}_i^\top \widehat{{\boldsymbol{\beta }}})\rbrace ]^2} \begin{bmatrix} 1 & {\boldsymbol{x}}_i^\top \\ {\boldsymbol{x}}_i & {\boldsymbol{x}}_i {\boldsymbol{x}}_i^\top \end{bmatrix}\,. \end{aligned}$$

Standard errors of parameter estimates are computed upon extracting the diagonal entries of $\lbrace {\boldsymbol{\mathcal {I}}}(\widehat{\beta }_0, \widehat{{\boldsymbol{\beta }}})\rbrace ^{-1}$, taking the square-root and multiplying it by $n^{-1/2}$.

### Related works

In the literature, computing $(\widehat{\beta }_0, \widehat{{\boldsymbol{\beta }}})$ and their standard errors in a vertically partitioned setting has been addressed in^8,9^, using an approach based on “secure sums” and “secure matrix product” algorithms. As mentioned in the introduction, this approach involves a high volume of communication between nodes and does not eliminate privacy risks, making it unsuitable for our intended applications.

Our approach is different, and approximates $(\widehat{\beta }_0, \widehat{{\boldsymbol{\beta }}})$ by solving a penalized version of (2), whose dual formulation enables computation in a vertically partitioned setting using a single round of communication. The penalty parameter is chosen to be sufficiently small so that the resulting estimates remain statistically equivalent to $(\widehat{\beta }_0, \widehat{{\boldsymbol{\beta }}})$.

Dual optimization for logistic regression with vertically partitioned data has previously been used in the VERTIGO algorithm^11^ and in the paper from our group revisiting the VERTIGO algorithm^14^, in the context of ridge regression (focusing on point estimates without CIs). The related VERTIGO-CI algorithm^12^ also uses this approach for logistic regression with CIs, also relying on a small penalty parameter as we do in this paper. However, aside from the fact that, as noted in the introduction, their approach allows for reverse-engineering of covariate-nodes data, the penalized version of (2) considered in that work differs from ours, and their methodology lacks the theoretical justification that we provide in Supplementary Methods for appropriately choosing $\lambda$.

In contrast to existing approaches based on dual optimization, our method does not require sharing the response vector across data nodes. Our procedure for enabling covariate-nodes to estimate their covariate-related parameters and compute their corresponding standard errors is therefore fundamentally different from all previously proposed methods.

It is worth mentioning that solving an approximation to the likelihood function in (2) from vertically partitioned data has been previously considered^25^, using an approach that does not rely on dual optimization, but instead employs an approximation to the sigmoid function combined with a series of additive secret-sharing operations. However, as previously noted^11^, this method entails high computational complexity and requires numerous communication rounds, which may be impractical for our intended real-world applications.

### Detailed steps of the VALORIS algorithm

Our algorithm VALORIS begins by requiring every covariate-node *k* to construct a centered and scaled version of the matrix, denoted by ${\boldsymbol{X}}^{(k)}_{\text {cs}}$. Every covariate-node then computes its local Gram matrix ${\boldsymbol{\mathcal {K}}}^{(k)} = {\boldsymbol{X}}^{(k)}_{\text {cs}} ({\boldsymbol{X}}^{(k)}_{\text {cs}})^\top$ and sends the obtained quantity to the response-node. Once the local Gram matrices have been received, the response-node numerically and locally solves the dual minimization problem given by

4 $$\begin{aligned} \min _{{\boldsymbol{\alpha }} \in (0,1)^n} \bigg ( J^\lambda ({{\boldsymbol{\alpha }}}) := \frac{1}{2\lambda n^2 } {{\boldsymbol{\alpha }}}^\top {{\,\textrm{diag}\,}}({{\boldsymbol{y}}})\Big ( \sum _{k=1}^K {\boldsymbol{\mathcal {K}}}^{(k)} + {\boldsymbol{1}}_{n}{\boldsymbol{1}}_{n}^\top \Big ){{\,\textrm{diag}\,}}({{\boldsymbol{y}}}) {{\boldsymbol{\alpha }}}+{\frac{1}{n}}\sum _{i=1}^n \Big \lbrace (1-\alpha _i)\log (1-\alpha _i)+ \alpha _i \log (\alpha _i) \Big \rbrace \bigg ), \end{aligned}$$

and obtains the dual estimates. The response-node subsequently sends to every covariate-node two node-specific intermediate quantities. The first intermediate quantity allows each covariate-node to solve a linear system of equations and retrieve the numerical estimates $\tilde{{\boldsymbol{\beta }}}^{\lambda (k)}$, corresponding to the components of $\tilde{{\boldsymbol{\beta }}}^\lambda$ associated with the covariates stored at that node. The second quantity allows for the local computation of associated standard errors without disclosing the response vector. The full VALORIS algorithm is stated in Algorithm 1.

**Algorithm 1:**
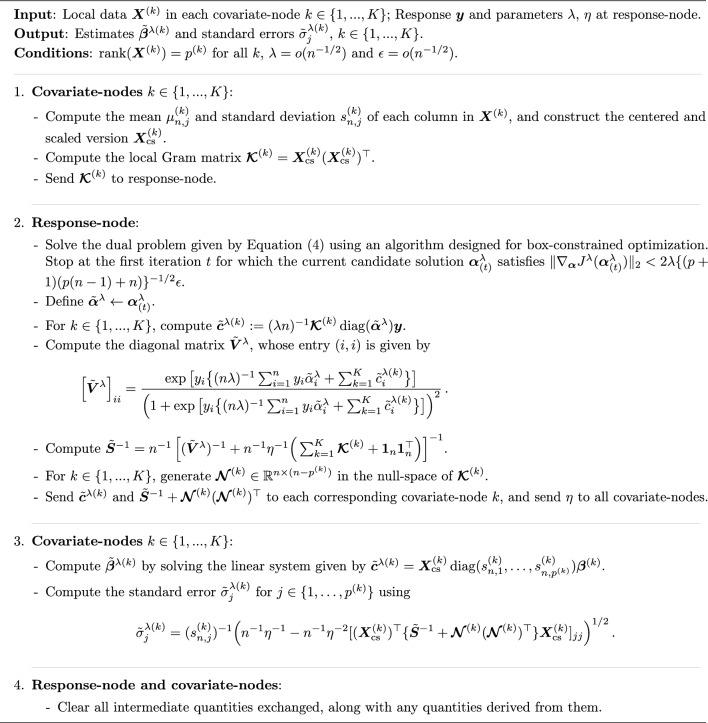
VALORIS: Vertically partitioned analytics under the lOgistic regression model for inference in statistics.

To solve (4), since the dual parameter ${\boldsymbol{\alpha }}$ is restricted to the domain $(0,1)^n$, our implementation employs the Two-Metric Projected Newton method^26,27^. The algorithm also takes as input a user-defined tolerance parameter $\epsilon> 0$, which defines the stopping criterion for the dual optimization procedure and ensures an upper bound on the difference between the numerical and the exact solutions (see Methods - *Methodology for the dual optimization program and stopping criterion in VALORIS*). Regarding parameters $\lambda$ and $\eta$, we experienced good numerical results with the choice $\lambda =5\times 10^{-6}$ and $\eta = 10^{-7}$ across various sample sizes, such that these values were set as default. Decreasing their values towards zero will narrow the difference between resulting estimates and their centralized counterpart, although this may require computations to be carried out at higher numerical precision. A sensitivity analysis with respect to the parameters $\lambda$, $\eta$ and $\epsilon$ was conducted, along with an illustrative case of strong response imbalance (2% of responses with $y_i=1$). The corresponding results, reported in Supplementary Tables S7 to S10, illustrate that for each of these parameters, there exists a range of values near the defaults used in our implementation for which the resulting approximation errors are comparable in magnitude to those obtained from the default settings, whereas choosing much smaller values for these parameters may adversely affect the behaviour of our implementation of the algorithm. Additionally, selected key implementation details for the VALORIS algorithm used in our implementation^18^ are provided in Supplementary Methods - *Implementation details and computational considerations of the VALORIS algorithm*, which also describe how the computational cost associated with our implementation scales with the sample size *n*, the number of sites *K*, and the number of covariates *p*.

We used $\tilde{{\boldsymbol{\alpha }}}^\lambda$ to denote the (approximate) numerical solution to (4) and we used a tilde to denote quantities computed from $\tilde{{\boldsymbol{\alpha }}}^\lambda$–such as $(\tilde{\beta }^\lambda _0, \tilde{{\boldsymbol{\beta }}}^\lambda )$, $\tilde{{\boldsymbol{c}}}^{(k)}$, $\tilde{{\boldsymbol{S}}}$, $\tilde{{\boldsymbol{V}}}^\lambda$, and $\tilde{\sigma }_j^{\lambda (k)}$. In the following Methods sections, we adopt a different notation to refer to the exact solution of (4), which will be denoted by $\widehat{{\boldsymbol{\alpha }}}^\lambda$, and we use a hat to indicate quantities derived from it (e.g., $(\widehat{\beta }^\lambda _0, \widehat{{\boldsymbol{\beta }}}^\lambda )$, $\widehat{{\boldsymbol{c}}}^{(k)}$, $\widehat{{\boldsymbol{S}}}$, $\widehat{{\boldsymbol{V}}}^\lambda$, and $\widehat{\sigma }_j^{\lambda (k)}$). We make this distinction to separate numerical approximations from theoretical quantities used in theoretical analyses, and to explicitly define and track the approximation error.

### Methodology for computing parameter estimates in VALORIS

To estimate $(\beta _{0\star }, {\boldsymbol{\beta }}_\star )$ in our vertically partitioned setting, we consider for $\lambda>0$ the following ridge-penalized logistic regression problem

5 $$\begin{aligned} \max _{\beta _0\in \mathbb {R},{\boldsymbol{\beta }}\in \mathbb {R}^p} \left( l^\lambda _n(\beta _0,{\boldsymbol{\beta }}) := \ell _n(\beta _0,{\boldsymbol{\beta }}) - \frac{\lambda }{2} \bigg [\big ( \beta _0+\sum _{j=1}^p\beta _j {\mu }_{n,j}\big )^2 + \sum _{j=1}^p{\beta _j^2}s_{n,j}^2 \bigg ]\right) \,. \end{aligned}$$

We denote $(\widehat{\beta }^\lambda _{0}, \widehat{{\boldsymbol{\beta }}}^\lambda ):= \arg \max _{\beta _0\in \mathbb {R},{\boldsymbol{\beta }}\in \mathbb {R}^p}l^\lambda _n(\beta _0,{\boldsymbol{\beta }})$, and we recall that ${\mu }_{n,j}$ and $s_{n,j}$ represent the mean and standard deviation of the *j*th column in ${\boldsymbol{X}}$, that is,

$$\begin{aligned} \mu _{n,j} = \frac{1}{n}\sum _{i=1}^n x_{ij}\,,\qquad \text{ and } \qquad s_{n,j}=\frac{1}{n-1}\sum _{i=1}^n (x_{ij}- \mu _{n,j})^2 \,. \end{aligned}$$

The vector ${\boldsymbol{x}}_{i,\text {cs}}$ denotes the centered and scaled covariates for observation *i*, with entries ${x}_{ij,\text {cs}} = (x_{ij} - {\mu }_{n,j}) / s_{n,j}$. It is shown in Supplementary Methods - *Equivalence to the standard non-penalized log-likelihood and computation of standard errors* that, under suitable and commonly satisfied assumptions, the absolute difference between $(\widehat{\beta }^\lambda _{0}, \widehat{{\boldsymbol{\beta }}}^\lambda )$ and the standard maximum likelihood estimator $(\widehat{\beta }_{0}, \widehat{{\boldsymbol{\beta }}})$ can be made arbitrarily small by choosing the penalty parameter sufficiently small. Equivalently, the precision of the proposed distributed estimator relative to the target centralized estimator can be made arbitrarily high as $\lambda \rightarrow 0$. The method therefore satisfies the lossless property for parameter estimation with respect to standard non-penalized logistic regression.

Recall $J^\lambda ({\boldsymbol{\alpha }})$ defined in (4), which represents the dual problem to the maximization problem in (5). For any $\lambda>0$, the solution $(\widehat{\beta }^\lambda _{0}, \widehat{{\boldsymbol{\beta }}}^\lambda )$ can be computed as

6 $$\begin{aligned} \widehat{\beta }^\lambda _0 = \frac{1}{n\lambda } \sum _{i=1}^n \widehat{\alpha }^\lambda _i y_i - \sum _{j=1}^p {\mu }_{n,j} \widehat{\beta }^\lambda _j \,, \quad \widehat{{\boldsymbol{\beta }}}^\lambda = {{\,\textrm{diag}\,}}\left( s_{n,1},\ldots , s_{n,p}\right) ^{-1}\left( \frac{1}{n\lambda } \sum _{i=1}^n \widehat{\alpha }^\lambda _i y_i {\boldsymbol{x}}_{i,\text {cs}}\right) \,, \end{aligned}$$

where $\widehat{{\boldsymbol{\alpha }}}^\lambda := [\widehat{\alpha }^\lambda _1, \ldots , \widehat{\alpha }^\lambda _n]^\top \in (0,1)^n$ is the unique minimizer of $J^\lambda ({\boldsymbol{\alpha }})$ over $(0,1)^n$ (see Supplementary Methods - *Details for the derivation of the dual optimization problem*).

As can be seen from the expression of $J^\lambda ({\boldsymbol{\alpha }})$, it depends on the covariate data only through $\sum _{k=1}^K{\boldsymbol{\mathcal {K}}}^{(k)}$, where ${\boldsymbol{\mathcal {K}}}^{(k)}={\boldsymbol{X}}^{(k)}_{\text {cs}}({\boldsymbol{X}}^{(k)}_{\text {cs}})^\top$ denotes the local Gram matrices. To solve (4) in our vertically partitioned setting, each covariate-node $k \in \{1, \ldots , K\}$ is required to compute and send the matrix ${\boldsymbol{\mathcal {K}}}^{(k)}$ to the response-node. The response-node can then solve the minimization problem on its own and obtain $\widehat{{\boldsymbol{\alpha }}}^\lambda$ (see details in Methods - *Methodology for the dual optimization program and stopping criterion in VALORIS*), as similarly done in previous work that required the response vector to be shared to all nodes^11,14^.

To enable each covariate-node to compute $\widehat{{\boldsymbol{\beta }}}^{\lambda (k)}$, where $\widehat{{\boldsymbol{\beta }}}^{\lambda (k)}$ denotes the $p^{(k)}$ components of $\widehat{{\boldsymbol{\beta }}}^\lambda$ corresponding to the covariates stored at node *k*, and so without access to the individual response values, we require the response-node to send a distinct vector to each covariate-node which allows to construct a system of equations whose unique solution is $\widehat{{\boldsymbol{\beta }}}^{\lambda (k)}$. Noting from expressing $\widehat{{\boldsymbol{\beta }}}^{\lambda (k)}$ in a vector-matrix notation that

$$\begin{aligned} {{\,\textrm{diag}\,}}( s^{(k)}_{n,1},\ldots , s^{(k)}_{n,p^{(k)}}) \widehat{{\boldsymbol{\beta }}}^{\lambda (k)}=(n\lambda )^{-1}({{\boldsymbol{X}}^{(k)}_{\text {cs}}})^{\top }{{\,\textrm{diag}\,}}(\widehat{{\boldsymbol{\alpha }}}^\lambda ){{\boldsymbol{y}}} \,, \end{aligned}$$

it follows that $\widehat{{\boldsymbol{\beta }}}^{\lambda (k)}$ satisfies

7 $$\begin{aligned} \begin{aligned} {{\boldsymbol{X}}^{(k)}_{\text {cs}}} {{\,\textrm{diag}\,}}( s^{(k)}_{n,1},\ldots , s^{(k)}_{n,p^{(k)}}) \widehat{{\boldsymbol{\beta }}}^{\lambda (k)}&= (n\lambda )^{-1}\big \lbrace {{\boldsymbol{X}}^{(k)}_{\text {cs}}}({{\boldsymbol{X}}^{(k)}_{\text {cs}}})^{\top }\big \rbrace {{\,\textrm{diag}\,}}(\widehat{{\boldsymbol{\alpha }}}^\lambda ){{\boldsymbol{y}}} \\&= (n\lambda )^{-1}{\boldsymbol{\mathcal {K}}}^{(k)} {{\,\textrm{diag}\,}}(\widehat{{\boldsymbol{\alpha }}}^\lambda ){{\boldsymbol{y}}} := \widehat{{\boldsymbol{c}}}^{\lambda (k)}\,. \end{aligned} \end{aligned}$$

If ${\boldsymbol{X}}^{(k)}_{\text {cs}}$ has full column rank, it follows that $\widehat{{\boldsymbol{\beta }}}^{\lambda (k)}$ is the unique solution of the system of equations

$$\begin{aligned} \widehat{{\boldsymbol{c}}}^{\lambda (k)} = {\boldsymbol{X}}^{(k)}_{\text {cs}} {{\,\textrm{diag}\,}}( s^{(k)}_{n,1},\ldots , s^{(k)}_{n,p^{(k)}}) {\boldsymbol{\beta }}^{(k)} \,, \ \ \ {\boldsymbol{\beta }}^{(k)} \in \mathbb {R}^{p^{(k)}}\,. \end{aligned}$$

### Methodology for computing standard errors of parameter estimates in VALORIS

In our vertically partitioned setting, the observed Fisher information matrix ${\boldsymbol{\mathcal {I}}}(\widehat{\beta }_0, \widehat{{\boldsymbol{\beta }}})$ defined in (3) cannot be directly computed. Alternatively, we show in Supplementary Methods - *Equivalence to the standard non-penalized log-likelihood and computation of standard errors* that, under the assumption that $\lambda$ is chosen to be sufficiently small, it holds for $j\in \lbrace 2,\ldots ,p+1\rbrace$ that $[ \lbrace {\boldsymbol{\mathcal {I}}}(\widehat{\beta }_0,\widehat{{\boldsymbol{\beta }}}) \rbrace ^{-1}]_{jj}$ is asymptotically equivalent to $[ ( {\boldsymbol{\mathcal {I}}}^\lambda )^{-1}]_{jj} / s_{n,j}^2\,$, with

$$\begin{aligned} {\boldsymbol{\mathcal {I}}}^\lambda&= n^{-1}\begin{bmatrix} {\boldsymbol{1}}_n&{\boldsymbol{X}}^{(1)}_{\text {cs}}&\ldots&{\boldsymbol{X}}^{(k)}_{\text {cs}}\end{bmatrix}^\top \widehat{{\boldsymbol{V}}}^\lambda \begin{bmatrix} {\boldsymbol{1}}_n&{\boldsymbol{X}}^{(1)}_{\text {cs}}&\ldots&{\boldsymbol{X}}^{(k)}_{\text {cs}}\end{bmatrix}, \end{aligned}$$

where $\widehat{{\boldsymbol{V}}}^\lambda$ is a diagonal matrix whose diagonal entries $[\widehat{{\boldsymbol{V}}}^\lambda ]_{jj}$ satisfy

$$\begin{aligned} {[}\widehat{{\boldsymbol{V}}}^\lambda ]_{jj}&= \frac{\exp \Big [ y_j\Big \lbrace (n\lambda )^{-1}\sum _{i=1}^n \widehat{\alpha }_i^\lambda y_i + \sum _{k=1}^K \widehat{{c}}^{\lambda (k)}_j\Big \rbrace \Big ] }{\Big ( 1+\exp \Big [ y_j\Big \lbrace (n\lambda )^{-1}\sum _{i=1}^n \widehat{\alpha }_i^\lambda y_i + \sum _{k=1}^K \widehat{{c}}^{\lambda (k)}_j\Big \rbrace \Big ] \Big )^2}\,. \end{aligned}$$

Therefore, when $\lambda$ is small, the standard error of each component *j* of $\widehat{{\boldsymbol{\beta }}}^\lambda$ can be consistently estimated using $s_{n,j}$ and the $(j+1)^\text {th}$ diagonal entry of $({\boldsymbol{\mathcal {I}}}^\lambda )^{-1}$.

To obtain $({\boldsymbol{\mathcal {I}}}^\lambda )^{-1}$, our approach builds on the Woodbury matrix identity^28^. This result states that, given a $p\times p$ invertible matrix ${\boldsymbol{A}}$, a $n \times n$ invertible matrix ${\boldsymbol{C}}$, and $p\times n$ matrices ${\boldsymbol{U}}$ and ${\boldsymbol{W}}$, if ${\boldsymbol{C}}^{-1}+{\boldsymbol{WA}}^{-1}{\boldsymbol{U}}$ is invertible, we have

$$\begin{aligned} ({\boldsymbol{A}}+{\boldsymbol{UCW}})^{-1}={\boldsymbol{A}}^{-1}-{\boldsymbol{A}}^{-1}{\boldsymbol{U}}({\boldsymbol{C}}^{-1}+{\boldsymbol{WA}}^{-1}{\boldsymbol{U}})^{-1}{\boldsymbol{WA}}^{-1}. \end{aligned}$$

For any $\eta>0$, and letting ${\boldsymbol{I}}_{r}$ denote the $r\times r$ identity matrix, the Woodbury matrix identity allows to express $\lbrace {\boldsymbol{\mathcal {I}}}^\lambda + \eta {\boldsymbol{I}}_{p+1} \rbrace ^{-1}$ as

8 $$\begin{aligned}&\Big ( {\boldsymbol{\mathcal {I}}}^\lambda + \eta {\boldsymbol{I}}_{p+1} \Big )^{-1} = \frac{1}{\eta }{\boldsymbol{I}}_{p+1}-\frac{1}{\eta ^2}\begin{bmatrix} {\boldsymbol{1}}_n^\top \widehat{{\boldsymbol{S}}}^{-1}{\boldsymbol{1}}_n & {\boldsymbol{1}}_n^\top \widehat{{\boldsymbol{S}}}^{-1}{{\boldsymbol{X}}^{(1)}_{\text {cs}}} & \cdots & {\boldsymbol{1}}_n^\top \widehat{{\boldsymbol{S}}}^{-1}{{\boldsymbol{X}}^{(K)}_{\text {cs}}} \\ ({{\boldsymbol{X}}^{(1)}_{\text {cs}}})^\top \widehat{{\boldsymbol{S}}}^{-1} {\boldsymbol{1}}_n& ({{\boldsymbol{X}}^{(1)}_{\text {cs}}})^\top \widehat{{\boldsymbol{S}}}^{-1}{{\boldsymbol{X}}^{(1)}_{\text {cs}}} & \cdots & ({{\boldsymbol{X}}^{(1)}_{\text {cs}}})^\top \widehat{{\boldsymbol{S}}}^{-1}{{\boldsymbol{X}}^{(K)}_{\text {cs}}} \\ \vdots & \vdots & \ddots & \vdots \\ ({{\boldsymbol{X}}^{(K)}_{\text {cs}}})^\top \widehat{{\boldsymbol{S}}}^{-1} {\boldsymbol{1}}_n & ({{\boldsymbol{X}}^{(K)}_{\text {cs}}})^\top \widehat{{\boldsymbol{S}}}^{-1}{{\boldsymbol{X}}^{(1)}_{\text {cs}}} & \cdots & ({{\boldsymbol{X}}^{(K)}_{\text {cs}}})^\top \widehat{{\boldsymbol{S}}}^{-1}{{\boldsymbol{X}}^{(K)}_{\text {cs}}} \end{bmatrix}\nonumber \,, \\&\text {where}\ \ \widehat{{\boldsymbol{S}}}:= \widehat{{\boldsymbol{S}}}(\eta ) = n(\widehat{{\boldsymbol{V}}}^\lambda )^{-1} + \frac{1}{\eta } \Big ( \sum _{k=1}^K {\boldsymbol{\mathcal {K}}}^{(k)} + {\boldsymbol{1}}_{n}{\boldsymbol{1}}_{n}^\top \Big ) \,. \end{aligned}$$

The parameter $\eta$ controls the accuracy of the approximation: as $\eta \rightarrow 0$, $({\boldsymbol{\mathcal {I}}}^{\lambda } + \eta {\boldsymbol{I}}_{p+1})^{-1}$ converges to $({\boldsymbol{\mathcal {I}}}^{\lambda })^{-1}$, allowing the approximation error to be made arbitrarily small as $\lambda$ is also taken sufficiently small, in accordance with the lossless property. In practice, the choice of $\eta$ may also be influenced by numerical considerations, as excessively small values can lead to instability at finite precision.

The diagonal entries of $\lbrace {\boldsymbol{\mathcal {I}}}^\lambda + \eta {\boldsymbol{I}}_{p+1} \rbrace ^{-1}$ can be extracted from the terms of the form $({{\boldsymbol{X}}^{(k)}_\textrm{cs}})^\top \widehat{{\boldsymbol{S}}}^{-1}{{\boldsymbol{X}}^{(k)}_\textrm{cs}}$. As the matrix $\widehat{{\boldsymbol{S}}}^{-1}$ can be entirely computed at the response-node, the latter computes and sends this quantity to each covariate-node *k* to allow them to obtain the diagonal entries of $({\boldsymbol{\mathcal {I}}}^\lambda + \eta {\boldsymbol{I}}_{p+1} )^{-1}$ corresponding to their covariate data by computing the diagonal entries of $({{\boldsymbol{X}}^{(k)}_\textrm{cs}})^\top \widehat{{\boldsymbol{S}}}^{-1}{{\boldsymbol{X}}^{(k)}_\textrm{cs}}$.

In addition, we note that, for any matrix ${\boldsymbol{\mathcal {N}}}^{(k)}\in \mathbb {R}^{n \times (n-p^{(k)})}$ in the null-space of $({{\boldsymbol{X}}^{(k)}_{\text {cs}}})^\top$ (i.e. such that $({{\boldsymbol{X}}^{(k)}_{\text {cs}}})^\top {\boldsymbol{\mathcal {N}}}^{(k)}={\boldsymbol{0}}_{p^{(k)}\times (n-p^{(k)})}$), it holds that $({{\boldsymbol{X}}^{(k)}_{\text {cs}}})^\top \widehat{{\boldsymbol{S}}}^{-1}{{\boldsymbol{X}}^{(k)}_{\text {cs}}}=({{\boldsymbol{X}}^{(k)}_{\text {cs}}})^\top \lbrace \widehat{{\boldsymbol{S}}}^{-1}+{\boldsymbol{\mathcal {N}}}^{(k)}({\boldsymbol{\mathcal {N}}}^{(k)})^\top \rbrace {{\boldsymbol{X}}^{(k)}_{\text {cs}}}$. The quantity ${\boldsymbol{\mathcal {N}}}^{(k)}$ can be generated at the response-node because the null-space of $({\boldsymbol{X}}^{(k)})^\top$ is the null-space of ${\boldsymbol{X}}^{(k)}({\boldsymbol{X}}^{(k)})^\top$^29^. Although $\widehat{{\boldsymbol{S}}}^{-1}$ could have been shared directly, we adopt a preventive approach by transmitting $\widehat{{\boldsymbol{S}}}^{-1}+{\boldsymbol{\mathcal {N}}}^{(k)}({\boldsymbol{\mathcal {N}}}^{(k)})^\top$ instead, to mitigate the risk of information leakage. Similar strategies for protecting exchanged quantities have been proposed in prior work, including^3^.

Covariate-node *k* can use the simplified expression in Algorithm 1 to compute the standard errors of the parameter estimate $\widehat{\beta }^{\lambda (k)}_j$ for $j\in \{1,\ldots , p^{(k)}\}$.

### Methodology for the dual optimization program and stopping criterion in VALORIS

The response-node solves the minimization problem in (4) to obtain $\tilde{{\boldsymbol{\alpha }}}^\lambda$ from which the $\tilde{{\boldsymbol{\beta }}}^{\lambda (k)}$’s are calculated. The details of the box-constrained convex optimization method used to compute $\tilde{{\boldsymbol{\alpha }}}^\lambda$ in our implementation are provided in Supplementary Methods - *Box-constrained optimization algorithm and stopping criteria*. The stopping criterion used in the VALORIS algorithm, which is independent of the specific optimization method employed, is given by

$$\begin{aligned} \Vert \nabla _{{{\boldsymbol{\alpha }}}} J^\lambda ({\tilde{{\boldsymbol{\alpha }}}^\lambda })\Vert _2 \le \frac{2\lambda }{\sqrt{p+1}} \Big ( p(n-1) + n \Big )^{-1/2} \epsilon \,, \end{aligned}$$

where the gradient of the dual objective function $J^\lambda ({\boldsymbol{\alpha }})$ is given by

$$\begin{aligned} \nabla _{{\boldsymbol{\alpha }}} J^\lambda ({\boldsymbol{\alpha }})=\frac{1}{\lambda n^2} {{\,\textrm{diag}\,}}({{\boldsymbol{y}}})\Big ( \sum _{k=1}^K {\boldsymbol{\mathcal {K}}}^{(k)} + {\boldsymbol{1}}_{n}{\boldsymbol{1}}_{n}^\top \Big ){{\,\textrm{diag}\,}}({{\boldsymbol{y}}}){{\boldsymbol{\alpha }}} + \frac{1}{ n}\left[ \log \left( \frac{\alpha _1}{1-\alpha _1}\right) , \cdots , \log \left( \frac{\alpha _n}{1-\alpha _n}\right) \right] ^\top . \end{aligned}$$

As we demonstrate in Supplementary Methods - *Box-constrained optimization algorithm and stopping criteria*, this entails the bound $\vert \widehat{\beta }^\lambda _j - \tilde{\beta }^\lambda _j \vert \le s^{-1}_{n,j}\epsilon$ for all $j \in \lbrace 1,\ldots , p\rbrace$.

### Methodology for assessing privacy-preserving properties

We carried a privacy analysis to ensure that individual-level data could not be reconstructed using reverse-engineering based on the notion of *admissible candidate datasets*–that is, admissible candidate matrices for covariate data and admissible candidate vectors for the response vector. For the purposes of the privacy analysis, we use the theoretical solution $\widehat{{\boldsymbol{\alpha }}}^\lambda$ (instead of its approximate solution $\tilde{{\boldsymbol{\alpha }}}^\lambda$) and its derived quantities. In addition to facilitating analytical derivations, this assumption provides an upper bound on potential privacy loss, as numerical implementations may introduce small perturbations that, in some cases, obscure exact reconstruction without eliminating the underlying risk. We also assume, as typically met in practice, that at least two covariate-nodes–including one potentially co-located at the response-node–participate in the analysis; for every individual $i\in \lbrace 1,\ldots , n\rbrace$, the scaled covariate values of this *i*th individual stored at node *k* are all different (i.e. $x_{ij,\text {cs}}^{(k)}\ne x_{ij',\text {cs}}^{(k)}$ for $j\ne j'$); none of the values are exactly equal to the associated-column mean (i.e. $x_{ij}^{(k)}\ne u_{n,j}^{(k)}$ for all *i*, *j*); the sample size is such that $p(p+3)/2 <n$; and there exists at least two observations such that $y_i=1$ and two such that $y_i=-1$.

#### Methodology to assess the privacy-preserving properties of quantities shared by a given covariate-node not co-located with the response-node

Considering a given covariate-node $k \in \{1, \ldots , K\}$, our analysis focuses on the potential reconstruction of the scaled data matrix ${\boldsymbol{X}}^{(k)}_{\text {cs}}$, even though access to ${\boldsymbol{X}}^{(k)}_{\text {cs}}$ does not necessarily permit recovery of the original data matrix ${\boldsymbol{X}}^{(k)}$. This methodological choice makes the results for continuous covariates more robust to adversarial nodes accessing external information regarding either covariates mean or covariates standard deviation, and thereby represents an additional layer of privacy protection.

We examine the set of quantities available to the response-node at a given point in time during the execution of Algorithm 1. We note that the response-node has temporary access to ${\boldsymbol{\mathcal {K}}}^{(k)}$ before Step 4 of the algorithm. After the algorithm has been run, the response-node has potential access to subset of the $\widehat{{\boldsymbol{\beta }}}^{\lambda (k)}$’s and the $\widehat{\sigma }^{\lambda (k)}_j$’s, depending on whether covariate-node *k* chooses to disclose them.

Our analysis aims to assess whether, when attempting to solve for ${\boldsymbol{X}}^{(k)}_{\text {cs}}$ using the disclosed quantities and available information, whenever applicable, the solution space–defined as the set of data matrices compatible with the available quantities or information–contains, for every entry of ${\boldsymbol{X}}^{(k)}_{\text {cs}}$, at least two *admissible candidate* matrices that differ at that entry. We define an admissible candidate matrix ${\boldsymbol{A}}$ for ${\boldsymbol{X}}^{(k)}_{\text {cs}}$ as one that satisfies the following two conditions: (a) the disclosed quantities could have been equivalently computed from ${\boldsymbol{A}}$ in place of ${\boldsymbol{X}}^{(k)}_{\text {cs}}$, in which case ${\boldsymbol{A}}$ is called a *candidate* matrix for ${\boldsymbol{X}}_{\text {cs}}^{(k)}$; and (b) there exists a matrix ${\boldsymbol{A}}_0$ such that for each $j \in \{1, \ldots , p^{(k)}\}$, all entries in column *j* of ${\boldsymbol{A}}_0$ lie in the support $\mathcal {D}^{(k)}_j$ of covariate *j*, e.g., $\{0,1\}$ for binary covariates and $\mathbb {R}$ for continuous ones, and such that ${\boldsymbol{A}}$ is a column-wise centered and scaled version of ${\boldsymbol{A}}_0$, in which case ${\boldsymbol{A}}$ is said to be *admissible*.

**When only the local Gram matrix is available to the response-node.** Let $\mathcal {M}_{n,p}(\mathbb {R})$ be the set of $n \times p$ real-valued matrices. When the local Gram matrix ${\boldsymbol{\mathcal {K}}}^{(k)}$ is the only quantity shared from covariate-node *k* to the response-node, reverse-engineering ${\boldsymbol{X}}^{(k)}_{\text {cs}}$ requires the response-node to solve for ${\boldsymbol{A}}\in \mathcal {M}_{n,p^{(k)}}(\mathbb {R})$ the system of equations ${\boldsymbol{\mathcal {K}}}^{(k)} = {\boldsymbol{A}} {\boldsymbol{A}}^\top$, under the constraints that ${\boldsymbol{A}}$ has empirical mean zero (i.e. ${\boldsymbol{A}}^\top {\boldsymbol{1}}_n = 0$) and empirical variance one (i.e. $\textrm{diag}_{\text {vec}}({\boldsymbol{A}}^\top {\boldsymbol{A}}) = (n-1) {\boldsymbol{1}}_{p^{(k)}}$, where $\textrm{diag}_{\text {vec}}({\boldsymbol{Z}})$ denotes the vector of diagonal entries of any square matrix ${\boldsymbol{Z}}$).

It can be directly verified that, as ${\boldsymbol{X}}^{(k)}_{\text {cs}}$ necessarily meets all requirements for such matrix ${\boldsymbol{A}}$, it follows that $-{\boldsymbol{X}}^{(k)}_{\text {cs}}$ also does. Whatever the nature (binary or continuous) of the covariates contained in ${\boldsymbol{X}}^{(k)}_{\text {cs}}$, $-{\boldsymbol{X}}^{(k)}_{\text {cs}}$ is an admissible candidate matrix because taking the negative values of the centered and scaled matrix preserves the nature of the covariate. Therefore, as $x^{(k)}_{ij,\text {cs}}\ne -x^{(k)}_{ij,\text {cs}}$ under the assumption that no value in ${\boldsymbol{X}}^{(k)}$ takes the exact value of its associated column mean, we always have two admissible candidate that differ for every entry.

While we do not expand on that matter here, in many settings, many more admissible candidate matrices, and even infinitely many, exist. This is explained by the fact that the product with an orthogonal matrix preserves the original Gram matrix. Indeed, consider ${\boldsymbol{P}} \in \mathcal {M}_{p^{(k)},p^{(k)}}(\mathbb {R})$ such that ${\boldsymbol{P}}{\boldsymbol{P}}^\top = {\boldsymbol{I}}_{p^{(k)}}$. It suffices to note that, since ${\boldsymbol{X}}^{(k)}_{\text {cs}}{\boldsymbol{X}}^{(k)\top }_{\text {cs}} = {\boldsymbol{\mathcal {K}}}^{(k)}$, we have $({\boldsymbol{X}}^{(k)}_{\text {cs}}{\boldsymbol{P}})({\boldsymbol{X}}^{(k)}_{\text {cs}}{\boldsymbol{P}})^\top = {\boldsymbol{X}}^{(k)}_{\text {cs}}({\boldsymbol{P}}{\boldsymbol{P}}^\top ) {\boldsymbol{X}}^{(k)\top }_{\text {cs}} = {\boldsymbol{X}}^{(k)}_{\text {cs}}{\boldsymbol{X}}^{(k)\top }_{\text {cs}}= {\boldsymbol{\mathcal {K}}}^{(k)}$. However, this does not ensure that ${\boldsymbol{X}}^{(k)}_{\text {cs}}{\boldsymbol{P}}$ is an adequate candidate for a scaled data matrix, nor that it can be taken as an admissible matrix in regards to the nature of the covariates.

**When only parameter estimates and their standard errors are available to the response-node, without the local Gram matrix and quantities derived from it.** This scenario is non-specific to our proposed method and arises in most vertically partitioned logistic regression when estimates and standard errors are publicly disclosed. While few information is available at the response-node under this scenario, investigations were conducted (see Supplementary Methods - *Privacy-preserving properties* for details). As a result, the condition $p(p+3)/2 <n$ ensures that sharing those estimates when the Gram matrix is unknown can be considered at least as private as sharing the Gram matrix to run the algorithm in the first place. Note that, in cases where all covariate-nodes–except possibly the one located at the response-node–contain only binary covariates, Privacy level II may not be attained. Indeed, due to the binary nature of the covariates unknown to the response-node, the response-node could theoretically enumerate all possible datasets formed by every combination of 0s and 1s for each entry. It could then identify those consistent with the quantities it possesses (i.e., parameter estimates or standard errors) by fitting the logistic regression model in (5) to each candidate dataset using the response vector it holds and comparing the resulting estimates to the disclosed values. In such cases, privacy may fail to hold, as it is possible that no two distinct datasets are consistent with the quantities available to the response-node. However, beyond the uncertainty regarding the uniqueness of the admissible candidate dataset, this procedure is computationally demanding.

#### Methodology to assess the privacy-preserving properties of quantities shared by the response-node

Consider a given covariate-node $k \in \{1, \ldots , K\}$ located outside of the response-node, assumed to be adversarial, and recall that the response-node shares with the latter the vector $\widehat{{\boldsymbol{c}}}^{\lambda (k)} = ({\lambda }n)^{-1}{\boldsymbol{\mathcal {K}}}^{(k)}{{\,\textrm{diag}\,}}(\widehat{{\boldsymbol{\alpha }}}^\lambda ){{\boldsymbol{y}}}$ and the matrix $\widehat{{\boldsymbol{S}}}^{-1}+{{\boldsymbol{\mathcal {N}}}}^{(k)}({{\boldsymbol{\mathcal {N}}}}^{(k)})^\top$, respectively. In the following analysis, we examine covariate-node *k*’s ability to reconstruct any entry of ${\boldsymbol{y}}$ based on $\widehat{{\boldsymbol{c}}}^{\lambda (k)}$ and $\widehat{{\boldsymbol{S}}}^{-1}+{{\boldsymbol{\mathcal {N}}}}^{(k)}({{\boldsymbol{\mathcal {N}}}}^{(k)})^\top$.

We assume that at least two covariate-nodes participate in the analysis (including the possibility that one is co-located with the response-node), that ${\boldsymbol{X}}^{(k)}$ has full-column rank, that $n>p^{(k)}$ and that at least one continuous covariate is held outside of covariate-node *k*.

First, $\widehat{{\boldsymbol{S}}}^{-1}$ cannot be retrieve from $\widehat{{\boldsymbol{S}}}^{-1}+{{\boldsymbol{\mathcal {N}}}}^{(k)}({{\boldsymbol{\mathcal {N}}}}^{(k)})^\top$ at covariate-node k, since

$$\begin{aligned} \widehat{{\boldsymbol{S}}}^{-1}+{{\boldsymbol{\mathcal {N}}}}^{(k)}({{\boldsymbol{\mathcal {N}}}}^{(k)})^\top =\lbrace \widehat{{\boldsymbol{S}}}^{-1}+{{\boldsymbol{\mathcal {N}}}}^{(k)}_2({{\boldsymbol{\mathcal {N}}}}^{(k)}_2)^\top \rbrace +\lbrace {{\boldsymbol{\mathcal {N}}}}^{(k)}{({\boldsymbol{\mathcal {N}}}}^{(k)})^\top -{{\boldsymbol{\mathcal {N}}}}^{(k)}_2({{\boldsymbol{\mathcal {N}}}}^{(k)}_2)^\top \rbrace , \end{aligned}$$

where ${\boldsymbol{\mathcal {N}}}_2$ is any other matrix selected in the null-space of $({\boldsymbol{X}}^{(k)})^\top$. Since $\lbrace \widehat{{\boldsymbol{S}}}^{-1} + {\boldsymbol{\mathcal {N}}}^{(k)}_2 ({\boldsymbol{\mathcal {N}}}^{(k)}_2)^\top \rbrace$ is symmetric and invertible (because $\widehat{{\boldsymbol{S}}}$ is symmetric positive-definite (as defined in (8)), and the sum of a positive-definite and a positive semi-definite matrix is itself positive-definite) while the matrix $\lbrace {\boldsymbol{\mathcal {N}}}^{(k)} ({\boldsymbol{\mathcal {N}}}^{(k)})^\top - {\boldsymbol{\mathcal {N}}}^{(k)}_2 ({\boldsymbol{\mathcal {N}}}^{(k)}_2)^\top \rbrace$ lies in the null space of $({\boldsymbol{X}}^{(k)}_{\text {cs}})^\top$, the values of $\widehat{{\boldsymbol{S}}}^{-1}$ cannot be recovered.

We now examine the ability of covariate-node *k* to retrieve the response vector ${\boldsymbol{y}}$ from $\widehat{{\boldsymbol{c}}}^{\lambda (k)}$. To do this, and recalling that covariate-node *k* has access to ${\boldsymbol{\mathcal {K}}}^{(k)}$ and to $\lambda$, let

$$\begin{aligned} \mathbb {S}(\widehat{{\boldsymbol{c}}}^{\lambda (k)}) = \lbrace {\boldsymbol{y}}^\dag \in \lbrace -1,1\rbrace ^n : \widehat{{\boldsymbol{c}}}^{\lambda (k)} = (n\lambda )^{-1} {\boldsymbol{\mathcal {K}}}^{(k)} {{\,\textrm{diag}\,}}({\boldsymbol{\alpha }}) {\boldsymbol{y}}^\dag \,\, \text {for some } {\boldsymbol{\alpha }}\in (0,1)^n\rbrace \,. \end{aligned}$$

To ensure sharing ${\boldsymbol{c}}^{(k)}$ preserves privacy, one needs to verify that, for $1\le i\le n$, there exists a vector ${\boldsymbol{y}}^{\dag (i)} \in \lbrace -1,1\rbrace ^n$ with ${y}^{\dag (i)}_i = - y_i$, such that ${\boldsymbol{y}}^{\dag (i)} \in \mathbb {S}(\widehat{{\boldsymbol{c}}}^{\lambda (k)})$. The goal is to ensure that every entry of the response vector can be flipped and still lead to an admissible candidate response vector. This can be done using linear programming algorithm designed to find feasible solutions under linear inequality constraints (see algorithm and details in Supplementary Methods - *Privacy-preserving properties*).

Finally, when a covariate-node is co-located with the response-node, an adversarial covariate-node could attempt to infer the covariate data held by the response-node. The reconstruction risk in this setting is conservatively assessed using the privacy analysis from when only parameter estimates and their standard errors are available to the response-node, without the local Gram matrix and quantities derived from it; the risk of retrieving the response-node’s covariate data is lower, since the adversarial covariate-node does not have access to ${\boldsymbol{y}}$. In the absence of continuous covariates held outside the adversarial covariate-node–i.e., when all other covariate-nodes contain only binary covariates–privacy may not be attained, since the adversarial node could theoretically enumerate all possible datasets, and in some cases, only one may be consistent with the quantities it possesses.

### Methodology for real health data applications

We applied the VALORIS algorithm detailed in Algorithm 1 to two cases involving real heath data and performed analyses in R (version 4.4.1)^21^. These real health data cases where selected to include both numerical and binary covariates, to represent cases with sample size respectively lower than 100 and higher than 10000, and to allow for varying number of covariate-nodes. Complete-case analyses were conducted, whereby individuals with missing values were excluded. When presented, Wald-type $1-\alpha$ CIs for the *j*th covariate were computed using $\tilde{\beta }_j^\lambda \pm z_{1-\alpha /2}\tilde{\sigma }_j^\lambda$. Computational details including wall-clock time and peak memory usage were reported in Supplementary Table S6. Experiments were run on a computer under Windows 11, with an Intel Core i7-11390H processor running at 3.40GHz, using 16GB of RAM. The reported measurements are necessarily dependent on the specific hardware and software environment; they are reported to provide indicative order-of-magnitude assessments rather than absolute performance guarantees.

The empirical criterion regarding the privacy-preserving properties for the response vector was verified (see Supplementary Methods - *Privacy-preserving properties*) and was met in both analyses, such that privacy was ensured for the response-node data.

The analysis using a de-identified clinical dataset from the Necker-Enfants Malades Hospital was performed in the context of the C’IL-LICO project. The C’IL-LICO project and study protocol received approval from the French National Ethics and Scientific Committee for Research, Studies and Evaluations in the Field of Health (CESREES) under the number #2201437. The data processing was approved by the French Data Protection Authority (CNIL) with a waiver of informed consent under number DR-2023- 017//920398v1. All methods were performed in accordance with the relevant guidelines and regulations.

## Supplementary Information

Supplementary Information.

## Data Availability

The synthetic dataset to test the implementation in R is publicly available on GitHub. The MIMIC-IV dataset is available online under certain conditions, including completion of mandatory training. The de-identified clinical dataset from the Necker-Enfants Malades Hospital used only as an example in this study is not publicly available following institutional officials recommendations. The code used in this study is available on GitHub: https://url.griis.ca/valoris.
