# Supplementary material for: VALORIS: One-shot and lossless vertical logistic regression for privacy-protecting multi-site health analytics
Source: Sci Rep. 2026 Mar 8;16:12558. doi: 10.1038/s41598-026-41936-y (PMC13086855; doi:10.1038/s41598-026-41936-y)
Supplement: Supplementary file 1 — Supplementary Information. [file 41598_2026_41936_MOESM1_ESM.pdf]

***VALORIS: One-shot and lossless vertical logistic regression for privacy-protecting multi-site health analytics* - Supplementary Information**

Supplementary Tables

- Tables S1-S3: We provide three notation glossaries: the first describes general notation conventions, the second describes quantities that pertain to our regression setting, and the last describes quantities that pertain to the vertical setting and the proposed algorithm.
- Tables S4-S5: We present the detailed estimates and standard errors obtained for the analysis using real health data pertaining first to chronic kidney disease (CKD), and second to the MIMIC-IV database.
- Table S6: We provide computational details pertaining to the three experiments that were run. Experiments were run on a computer under Windows 11, with an Intel Core i7-11390H processor running at 3.40GHz, using 16GB of RAM.
- Tables S7-S9: We provide a sensitivity analysis for the method parameters (λ, η and ϵ), ran using the simulated dataset available on the Git repertory ($n = 300$).
- Table S10: We provide a case of response imbalance, where the simulated dataset available on the Git repertory ($n = 300$) was modified such that only the first 2% ($n = 6$) of individuals for which the response was 1 were left unchanged, and all others were set at $y = -1$.

Table S1. Glossary for general notation conventions

Random variable in $\mathbb{R}$	A	Uppercase Non-italic
Random vector in $\mathbb{R}^p$	$\mathbf{A}$	Uppercase Non-italic bold
Scalar in $\mathbb{R}$	a	Lowercase Italic
Vector in $\mathbb{R}^p$	$\boldsymbol{a}$	Lowercase Italic bold
Vector in $\mathbb{R}^p$ with all components equal to 1	$\mathbf{1}_p$	-
Matrix in $\mathbb{R}^{n \times p}$	$\boldsymbol{A}$	Uppercase Italic bold
Identity matrix in $\mathbb{R}^{n \times n}$	$\boldsymbol{I_n}$	-
Gradient of $f(\boldsymbol{\theta})$ (column vector)	$\nabla_{\boldsymbol{\theta}} f(\boldsymbol{\theta})$	$\nabla^2_{\boldsymbol{\theta}}$ for Hessian
$\nabla_{\boldsymbol{\theta}} f(\boldsymbol{\theta})\|_{\boldsymbol{\theta}=\boldsymbol{a}}$	$\nabla_{\boldsymbol{\theta}} f(\boldsymbol{a})$	$\nabla^2_{\boldsymbol{\theta}} f(\boldsymbol{a})$ for Hessian
$\max_{1 \leq j \leq p} \|a_j\|$	$\|\|\boldsymbol{a}\|\|_\infty$	Infinite norm
$\sum_{j=1}^{p} \|a_j\|$	$\|\|\boldsymbol{a}\|\|_1$	ℓ1-norm
$\sqrt{\sum_{j=1}^{p} a_i^2}$	$\|\|\boldsymbol{a}\|\|_2$	ℓ2-norm
Diagonal matrix with entries of $\boldsymbol{a}$ on diagonal	$\mathrm{diag}(\boldsymbol{a})$	Dimension $p \times p$ for $\boldsymbol{a} \in \mathbb{R}^p$
Quantity $\cdot$ at iteration t (step count)	$\cdot_{(t)}$	Starts with $\cdot_{(0)}$

Table S2. Glossary for quantities that pertain to the regression settings

Covariate vector for *ith* individual	$\boldsymbol{x}_i = [x_{i1}, \ldots, x_{ip}]^\top$
Covariate vector for *ith* individual with intercept	$[1, \boldsymbol{x}_i^\top]^\top$
Covariate matrix in $\mathbb{R}^{n \times p}$	$\boldsymbol{X} = \begin{bmatrix} x_{11} & \cdots & x_{1p} \\ \vdots & \ddots & \vdots \\ x_{n1} & \cdots & x_{np} \end{bmatrix} = \begin{bmatrix} \boldsymbol{x}_1^\top \\ \vdots \\ \boldsymbol{x}_n^\top \end{bmatrix}$
True (unknown) parameters	$\beta_{0\star}, \boldsymbol{\beta}_\star$
Exact MLE of the parameter	$\widehat{\beta}_0, \widehat{\boldsymbol{\beta}}$
Exact penalized estimate of the parameter	$\widehat{\beta}_0^\lambda, \widehat{\boldsymbol{\beta}}^\lambda$
Estimate obtained via numerical approximation	$\tilde{\beta}_0, \tilde{\boldsymbol{\beta}}, \tilde{\beta}_0^\lambda, \tilde{\boldsymbol{\beta}}^\lambda$
Log-likelihood	$\ell_n(\boldsymbol{\beta}) = \frac{1}{n}\sum_{i=1}^n \log(\cdot)$
Penalized log-likelihood	$l_n^\lambda(\boldsymbol{\beta})$
Mean of the jth column in covariate matrix	$u_{n,j}$
Standard deviation of the jth column in covariate matrix	$s_{n,j}$
Fisher information matrix	$\boldsymbol{\mathcal{I}}(\beta_0, \boldsymbol{\beta})$

Table S3. Glossary for quantities specific to the vertical setting

Number of covariate-nodes	K
Number of covariates at covariate-node k	$p^{(k)}$
Covariate matrix at covariate-node k	$\boldsymbol{X}^{(k)}$
Centered and scaled covariate matrix at covariate-node k	$\boldsymbol{X}_{\text{cs}}^{(k)}$
Mean and standard deviation of the jth column in $\boldsymbol{X}^{(k)}$	$u_{n,j}^{(k)}, s_{n,j}^{(k)}$
Gram matrix at covariate-node k	$\boldsymbol{\mathcal{K}}^{(k)} := \boldsymbol{X}_{\text{cs}}^{(k)} (\boldsymbol{X}_{\text{cs}}^{(k)})^\top$
Dual parameter estimates (numerical approximation)	$\tilde{\boldsymbol{\alpha}}^\lambda$
Penalized estimate associated with covariate-node k (numerical approximation)	$\tilde{\beta}_j^{\lambda(k)}$
Standard errors associated with covariate-node k (numerical approximation)	$\tilde{\sigma}_j^{\lambda(k)}$
Matrix in null-space of $\boldsymbol{\mathcal{K}}^{(k)}$	$\boldsymbol{\mathcal{N}}^{(k)}$
Intermediate quantities	$\tilde{\boldsymbol{c}}^{\lambda(k)}$, $\tilde{\boldsymbol{S}}$

Table S4. Logistic regression model parameter estimates obtained with kidney failure at two years after baseline as outcome

Covariate-node	Variable	Vertical Partition $\tilde{\beta}^\lambda(\tilde{\sigma}^\lambda)$	Centralized $\widehat{\beta}(\widehat{\sigma})$
1	Age	0.0509(0.0594)	0.0509(0.0594)
1	Sex (male)	−0.7731(0.5336)	−0.7732(0.5337)
1	eGFR	−0.0076(0.0270)	−0.0076(0.0270)
1	uACR	−0.2069(0.1610)	−0.2069(0.1609)
2	Serum albumin	−0.6463(0.4388)	−0.6464(0.4389)
2	Serum bicarbonate	−0.0997(0.0806)	−0.0997(0.0806)
2	Serum calcium	0.3463(0.5243)	0.3464(0.5242)
2	Serum phosphate	0.5202(0.2888)	0.5202(0.2887)

A log-transformation was applied to uACR due to a skewed distribution[1].
The estimates for the centralized setting were computed using the *glm* function in R with a non-penalized logistic regression model.

Table S5. Logistic regression model parameter estimates obtained with MIMIC-IV database and *Death* as outcome

Covariate-node	Variable	Vertical Partition $\tilde{\beta}^{\lambda}(\tilde{\sigma}^{\lambda})$	Centralized $\widehat{\beta}(\widehat{\sigma})^1$
1	Age	0.0272(0.0023)	0.0272(0.0023)
1	Myocardial infarction	0.4052(0.0828)	0.4052(0.0828)
1	Renal disease	0.1867(0.0751)	0.1867(0.0751)
1	Malignant cancer	0.8814(0.0716)	0.8815(0.0716)
2	Triage temperature	−0.0951(0.0276)	−0.0951(0.0276)
2	Triage sbp	−0.0033(0.0015)	−0.0033(0.0015)
2	Triage dbp	−0.0097(0.0025)	−0.0097(0.0025)
2	Triage respiratory rate > 20	0.4752(0.0856)	0.4752(0.0856)
3	First-day urine output	−0.0005(0.0000)	−0.0005(0.0000)
3	First-day heart rate mean	0.0319(0.0020)	0.0319(0.0020)
3	First-day glucose mean	0.0025(0.0006)	0.0025(0.0006)

[1]The estimates for the centralized setting were computed using the *glm* function in R with a non-penalized logistic regression model.

Table S6. Running time, Peak RAM used and Message size for numerical experiments

		Necker Hospital dataset ($n = 97$)	Simulated dataset ($n = 300$)	MIMIC-IV dataset ($n = 13677$)[1]
Wall-clock time (seconds)	Response-node	0.53	0.56	89.81
	Covariate-node	0.08	0.09	22.44
Peak RAM used (MiB)	Response-node	1.2	4.0	5712.3
	Covariate-node	0.9	2.2	2146.2
Total message size (kB)	From response-node (two files)	2.19 + 36.52	6.59 + 346.97	311.36 + 719592.59
	From covariate-node	36.94	350.09	720425.81

[1]For the case of multiple covariate-nodes not co-located at the response-node, the maximum values observed were reported.

Table S7. Sensitivity analysis with respect to the parameter λ, with fixed $\eta = 10^{-7}$ and $\epsilon = n^{-1}$ (default values)

	λ			
	5×10^{-6} **(default)**	10^{-6}	5×10^{-7}	10^{-7}
Max. abs. difference in parameter estimates [1]	1.85×10^{-4}	3.71×10^{-5}	1.85×10^{-5}	Does not terminate[2]
Max. abs. difference in standard error estimates [1]	5.44×10^{-5}	6.96×10^{-5}	5.48×10^{-5}	Does not terminate[2]

[1]Maximum absolute difference between the obtained estimates with VALORIS and those obtained with the glm function in R for the standard non-penalized logistic regression.

[2]The stopping criteria over the norm of the gradient for the optimization procedure depends on the value of λ(and the tolerance parameter ϵ), which ensures that theoretical properties are met (see Supplementary Methods – *Box-constrained optimization algorithm and stopping criteria*). Sensitivity analysis for ϵ is provided below; a higher choice for ϵ could allow convergence for lower values for λ.

Table S8. Sensitivity analysis with respect to the parameter η, with fixed $\lambda = 5 \times 10^{-6}$ and $\epsilon = n^{-1}$ (default values)

	η				
	10^{-5}	10^{-6}	10^{-7} (**default**)	10^{-8}	10^{-9}
Max. abs. difference in parameter estimates [1]	1.85×10^{-4}	1.85×10^{-4}	1.85×10^{-4}	1.85×10^{-4}	1.85×10^{-4}
Max. abs. difference in standard error estimates [1]	4.19×10^{-5}	2.40×10^{-5}	5.44×10^{-5}	4.16×10^{-3}	NA produced

[1]Maximum absolute difference between the obtained estimates with VALORIS and those obtained with the glm function in R for the standard non-penalized logistic regression.

Table S9. Sensitivity analysis with respect to the parameter ϵ, parametrized here as $\epsilon = hn^{-1}$ for different choices of h, with fixed $\lambda = 5 \times 10^{-6}$ and $\eta = 10^{-7}$ (default values)

	h				
	0.1	0.5	1 (**default**)	5	10
Max. abs. difference in parameter estimates [1]	1.85×10^{-4}	1.85×10^{-4}	1.85×10^{-4}	1.85×10^{-4}	1.85×10^{-4}
Max. abs. difference in standard error estimates [1]	5.44×10^{-5}	5.44×10^{-5}	5.44×10^{-5}	5.44×10^{-5}	5.44×10^{-5}

[1]Maximum absolute difference between the obtained estimates with VALORIS and those obtained with the glm function in R for the standard non-penalized logistic regression.

Table S10. Response imbalance over simulated dataset (with 6 over 300 responses at $y = 1$)

Max. abs. difference in parameter estimates [1]	2.84×10^{-3}
Mean abs. difference in parameter estimates [1]	1.18×10^{-3}
Max. abs. difference in standard error estimates [1]	9.64×10^{-4}
Mean abs. difference in standard error estimates [1]	5.60×10^{-4}

[1]Maximum and mean absolute difference between the obtained estimates with VALORIS and those obtained with the glm function in R for the standard non-penalized logistic regression.

Supplementary Notes

The code for the implementation of the algorithm using R is available at: https://url.griis.ca/valoris. It includes an automated example with simulated data. The folder also includes a basic implementation of the tool that supports the privacy assessment for the response-node individual-level data.

Supplementary Methods - Details for the derivation of the dual optimization problem

Recall from the manuscript that to estimate $(\beta_{0\star}, \boldsymbol{\beta}_\star)$, we consider the solutions $(\widehat{\beta}_0^\lambda, \widehat{\boldsymbol{\beta}}^\lambda)$ of the ridge-penalized logistic regression problem

$$\max_{\beta_0\in\mathbb{R},\boldsymbol{\beta}\in\mathbb{R}^p} \left(l_n^\lambda(\beta_0, \boldsymbol{\beta}) = \ell_n(\beta_0, \boldsymbol{\beta}) - \frac{\lambda}{2}\left[\left(\beta_0 + \sum_{j=1}^p \beta_j \mu_{n,j}\right)^2 + \sum_{j=1}^p \beta_j^2 s_{n,j}^2\right]\right),$$

where

$$\ell_n(\beta_0, \boldsymbol{\beta}) = n^{-1} \sum_{i=1}^n \log\left[\frac{1}{1+\exp\{-y_i(\beta_0 + \boldsymbol{x}_i^\top \boldsymbol{\beta})\}}\right].$$

Since, for all $i \in \{1, \dots, n\}$ we have

$$\begin{aligned}
\beta_0 + \boldsymbol{x}_i^\top \boldsymbol{\beta} &= \beta_0 + \sum_{j=1}^p x_{ij}\beta_j \\
&= \left(\beta_0 + \sum_{j=1}^p \mu_{n,j}\beta_j\right) + \sum_{j=1}^p \left(\frac{x_{ij} - \mu_{n,j}}{s_{n,j}}\right)(\beta_j s_{n,j}) \\
&= \left(\beta_0 + \widehat{\boldsymbol{\mu}}^\top \boldsymbol{\beta}\right) + \boldsymbol{x}_{i,\text{cs}}^\top \widehat{\boldsymbol{\Sigma}} \boldsymbol{\beta},
\end{aligned}$$

where we have introduced

$$\widehat{\boldsymbol{\mu}} = [\mu_{n,1}, \dots, \mu_{n,p}]^\top, \qquad \widehat{\boldsymbol{\Sigma}} = \text{diag}([s_{n,1}, \dots, s_{n,p}]^\top), \tag{S1}$$

it follows upon adopting the re-parametrization $(\beta_0^\circ, \boldsymbol{\beta}^\circ) \equiv (\beta_0 + \widehat{\boldsymbol{\mu}}^\top \boldsymbol{\beta}, \widehat{\boldsymbol{\Sigma}}\boldsymbol{\beta})$ that

$$\begin{aligned}
&\max_{\beta_0\in\mathbb{R},\boldsymbol{\beta}\in\mathbb{R}^p} l_n^\lambda(\beta_0, \boldsymbol{\beta}) \\
&= \max_{\beta_0^\circ\in\mathbb{R},\boldsymbol{\beta}^\circ\in\mathbb{R}^p} \left(\check{l}_n^\lambda(\beta_0^\circ, \boldsymbol{\beta}^\circ) := \check{\ell}_n(\beta_0^\circ, \boldsymbol{\beta}^\circ) - \frac{\lambda}{2}\sum_{j=0}^p (\beta_j^\circ)^2\right), \\
&\qquad \text{with } \check{\ell}_n(\beta_0^\circ, \boldsymbol{\beta}^\circ) := -n^{-1}\sum_{i=1}^n \log\left[1 + \exp\{-y_i(\beta_0^\circ + \boldsymbol{x}_{i,\text{cs}}^\top \boldsymbol{\beta}^\circ)\}\right].
\end{aligned}$$

Following the derivations from Li et al.[2] (see Appendix A therein), since the concave dual conjugate of $x \mapsto (1+e^{-x})^{-1}$ is $y \mapsto y\log y + (1-y)\log(1-y)$ for $y \in (0,1)$, we obtain that the Fenchel dual of $\check{l}_n^\lambda(\beta_0^\circ, \boldsymbol{\beta}^\circ)$ is the following minimization problem:

$$\begin{aligned}
\min_{\boldsymbol{\alpha}\in(0,1)^n} &\left(\frac{1}{n}\sum_{i=1}^n \left\{(1-\alpha_i)\log(1-\alpha_i) + \alpha_i \log(\alpha_i)\right\}\right. \\
&\left. + \frac{1}{2\lambda n^2}\sum_{i=1}^n\sum_{j=1}^n \alpha_i\alpha_j y_i y_j (\boldsymbol{x}_{i,\text{cs}}^\top \boldsymbol{x}_{j,\text{cs}} + 1)\right) = \min_{\boldsymbol{\alpha}\in(0,1)^n} J^\lambda(\boldsymbol{\alpha}).
\end{aligned}$$

Its unique maximizer $\widehat{\boldsymbol{\alpha}}^\lambda = (\widehat{\alpha}_1^\lambda, \dots, \widehat{\alpha}_n^\lambda)^\top \in (0,1)^n$ then satisfies

$$\begin{bmatrix}\check{\beta}_0^\lambda \\ \check{\boldsymbol{\beta}}^\lambda\end{bmatrix} = (n\lambda)^{-1}\sum_{i=1}^n y_i \widehat{\alpha}_i^\lambda \begin{bmatrix} 1 \\ \boldsymbol{x}_{i,\text{cs}}\end{bmatrix}.$$

Since the bijective nature of the reparametrization implies

$$\begin{bmatrix}\widehat{\beta}_0^\lambda \\ \widehat{\boldsymbol{\beta}}^\lambda\end{bmatrix} = \begin{bmatrix}\check{\beta}_0^\lambda - \widehat{\boldsymbol{\mu}}^\top \widehat{\boldsymbol{\beta}}^\lambda \\ \widehat{\boldsymbol{\Sigma}}^{-1}\check{\boldsymbol{\beta}}^\lambda\end{bmatrix}, \tag{S2}$$

we derive that

$$\widehat{\beta}_0^\lambda = (n\lambda)^{-1}\sum_{i=1}^n y_i\widehat{\alpha}_i^\lambda - \widehat{\boldsymbol{\mu}}^\top\widehat{\boldsymbol{\beta}}^\lambda, \qquad \widehat{\boldsymbol{\beta}}^\lambda = \widehat{\boldsymbol{\Sigma}}^{-1}\left((n\lambda)^{-1}\sum_{i=1}^n y_i\widehat{\alpha}_i^\lambda \boldsymbol{x}_{i,\mathrm{cs}}\right).$$

By additionally noting that $\check{l}_n^\lambda(\beta_0, \boldsymbol{\beta})$ is strongly concave, its maximum is unique and is achieved at the point $(\check{\beta}_0^\lambda, \check{\boldsymbol{\beta}}^\lambda)$ that satisfies

$$n^{-1}\sum_{i=1}^n \frac{y_i}{1+\exp\{y_i(\check{\beta}_0^\lambda + \boldsymbol{x}_i^\top\check{\boldsymbol{\beta}}^\lambda)\}}\begin{bmatrix}1\\ \boldsymbol{x}_i\end{bmatrix} = \lambda\begin{bmatrix}\check{\beta}_0^\lambda \\ \check{\boldsymbol{\beta}}^\lambda\end{bmatrix},$$

we can use the triangle inequality and derive

$$\max_{0\le j\le p}|\check{\beta}_j^\lambda| \le \lambda^{-1}\Big(n^{-1}\sum_{i=1}^n(\|\boldsymbol{x}_i\|_\infty + 1)\Big). \tag{S3}$$

Therefore, the primal maximization problem can be restricted over a compact search space, and the relation between the dual and primal parameters implies that the dual maximization problem can also be restricted over a compact search space.

Supplementary Methods - Equivalence to the standard non-penalized log-likelihood and computation of standard errors

Equivalence between parameter estimates

In this section, we formally establish, under appropriate conditions, the equivalence between the penalized estimators $(\widehat{\beta}_0^\lambda, \widehat{\boldsymbol{\beta}}^\lambda)$, which maximize $\ell_n^\lambda(\beta_0, \boldsymbol{\beta})$, and the unpenalized maximum likelihood estimators. To ensure this equivalence, the conditions must guarantee that maximum likelihood estimates exist and are unique. It is well known[3] that if the columns of $\boldsymbol{X}$ are linearly independent and also linearly independent of the vector $\mathbf{1}_n$, then the Hessian $\nabla^2_{\beta_0,\boldsymbol{\beta}}\ell_n(\beta_0, \boldsymbol{\beta})$ is strictly negative definite, which implies that the log-likelihood function $\ell_n(\beta_0, \boldsymbol{\beta})$ is strictly concave. In this case, if a maximizer exists for the problem $\max_{\beta_0\in\mathbb{R},,\boldsymbol{\beta}\in\mathbb{R}^p}\ell_n(\beta_0, \boldsymbol{\beta})$, then it must be unique and coincide with a stationary point of $\nabla_{\beta_0,\boldsymbol{\beta}}\ell_n(\beta_0, \boldsymbol{\beta})$. The existence of such a solution is guaranteed when the response vector $\boldsymbol{y}$ is not separable[4]. Specifically, $\boldsymbol{y}$ is said to be separable if there exists $(\beta_0, \boldsymbol{\beta})$ such that $y_i(\beta_0 + \boldsymbol{x}_i^\top\boldsymbol{\beta}) > 0$ for all $i \in 1, \dots, n$. We will assume these conditions throughout the following discussion. Let $(\widehat{\beta}_0, \widehat{\boldsymbol{\beta}})$ denote the unique maximizer of $\ell_n(\beta_0, \boldsymbol{\beta})$.

In what follows, for any positive definite matrix $\boldsymbol{A}$, let $\iota_{\min}(\boldsymbol{A})$ denote its smallest eigen value. Also, let $M \ge 1$ be a constant such that $\max_{1\le i\le n}\|\boldsymbol{x}_{i,\mathrm{cs}}\|_\infty \le M$.

To establish the statistical equivalence between $(\widehat{\beta}_0^\lambda, \widehat{\boldsymbol{\beta}}^\lambda)$ and $(\widehat{\beta}_0, \widehat{\boldsymbol{\beta}})$, we start by proving an inequality that involves the reparametrized version of $(\widehat{\beta}_0^\lambda, \widehat{\boldsymbol{\beta}}^\lambda)$ introduced in Supplementary Methods - *Details for the derivation of the dual optimization problem*, namely $(\check{\beta}_0^\lambda, \check{\boldsymbol{\beta}}^\lambda) = \arg\max_{\beta_0\in\mathbb{R},\boldsymbol{\beta}\in\mathbb{R}^p}\check{l}_n^\lambda(\beta_0, \boldsymbol{\beta})$, and a corresponding reparametrized version of the maximum likelihood estimates $(\widehat{\beta}_0, \widehat{\boldsymbol{\beta}})$. Specifically, by reparametrisation arguments analogous to those in the previous Supplementary Methods, it follows that $\max_{\beta_0\in\mathbb{R},\boldsymbol{\beta}\in\mathbb{R}^p}\ell_n(\beta_0, \boldsymbol{\beta}) = \max_{\beta_0^\circ\in\mathbb{R},\boldsymbol{\beta}^\circ\in\mathbb{R}^p}\check{\ell}_n(\beta_0^\circ, \boldsymbol{\beta}^\circ)$, with

$\check{\ell}_n$ defined in the previous Supplementary Methods. Moreover, $\check{\ell}_n$ has a unique maximizer $(\check{\beta}_0, \check{\boldsymbol{\beta}})$ satisfying

$$\begin{bmatrix} \widehat{\beta}_0 \\ \widehat{\boldsymbol{\beta}} \end{bmatrix} = \begin{bmatrix} \check{\beta}_0 - \widehat{\boldsymbol{\mu}}^\top \widehat{\boldsymbol{\beta}} \\ \widehat{\boldsymbol{\Sigma}}^{-1} \check{\boldsymbol{\beta}} \end{bmatrix} .$$

To derive the inequality, we adopt some notational simplifications. We will occasionally write $\boldsymbol{z}_i = [1, \boldsymbol{x}_i^\top]^\top$ and $\boldsymbol{z}_{i,\mathrm{cs}} = [1, \boldsymbol{x}_{i,\mathrm{cs}}^\top]^\top$. We will also use the shorthand $\widehat{\boldsymbol{\theta}}^\lambda$, $\widehat{\boldsymbol{\theta}}$, $\check{\boldsymbol{\theta}}^\lambda$, and $\check{\boldsymbol{\theta}}$ to denote, respectively, $[\widehat{\beta}_0^\lambda, (\widehat{\boldsymbol{\beta}}^\lambda)^\top]^\top$, $[\widehat{\beta}_0, \widehat{\boldsymbol{\beta}}^\top]^\top$, $[\check{\beta}_0^\lambda, (\check{\boldsymbol{\beta}}^\lambda)^\top]^\top$, and $[\check{\beta}_0, \check{\boldsymbol{\beta}}^\top]^\top$. In this notation, the inequality we shall prove below is the following:

$$\|\check{\boldsymbol{\theta}}^\lambda - \check{\boldsymbol{\theta}}\|_\infty \leq \frac{(p+1)\lambda \|\check{\boldsymbol{\theta}}\|_\infty}{\lambda + \omega\{(p+2)^2 \|\check{\boldsymbol{\theta}}\|_\infty\} \iota_{\min}(n^{-1} \sum_{i=1}^n \boldsymbol{z}_{i,\mathrm{cs}} \boldsymbol{z}_{i,\mathrm{cs}}^\top)} , \tag{S4}$$

where $\omega(x) := e^x/(1+e^x)^2$.

Since $\check{\boldsymbol{\theta}}^\lambda$ and $\check{\boldsymbol{\theta}}$ are the unique stationary points of $\check{\ell}_n^\lambda$ and $\check{\ell}_n$, respectively, they satisfy the corresponding first-order conditions, that is, they cancel the gradients of these functions. Hence, we have

$$\begin{aligned} \lambda \check{\boldsymbol{\theta}}^\lambda &= n^{-1} \sum_{i=1}^n y_i \Big[\frac{1}{1 + \exp(y_i \boldsymbol{z}_{i,\mathrm{cs}}^\top \check{\boldsymbol{\theta}}^\lambda)} - \frac{1}{1 + \exp(y_i \boldsymbol{z}_{i,\mathrm{cs}}^\top \check{\boldsymbol{\theta}})} \Big] \boldsymbol{z}_{i,\mathrm{cs}} \\ &= n^{-1} \sum_{i=1}^n y_i \Big[\frac{1}{1 + \exp\{y_i \boldsymbol{z}_{i,\mathrm{cs}}^\top (\check{\boldsymbol{\theta}} + \boldsymbol{\Delta})\}} - \frac{1}{1 + \exp(y_i \boldsymbol{z}_{i,\mathrm{cs}}^\top \check{\boldsymbol{\theta}})} \Big] \boldsymbol{z}_{i,\mathrm{cs}} , \end{aligned}$$

where we have introduced $\boldsymbol{\Delta} = \check{\boldsymbol{\theta}}^\lambda - \check{\boldsymbol{\theta}}$. Since

$$\frac{1}{1 + \exp\{y_i \boldsymbol{z}_{i,\mathrm{cs}}^\top (\check{\boldsymbol{\theta}} + \boldsymbol{\Delta})\}} - \frac{1}{1 + \exp(y_i \boldsymbol{z}_{i,\mathrm{cs}}^\top \check{\boldsymbol{\theta}})} = -\int_0^1 \frac{\exp\{y_i \boldsymbol{z}_{i,\mathrm{cs}}^\top (\check{\boldsymbol{\theta}} + t\boldsymbol{\Delta})\}}{[1 + \exp\{y_i \boldsymbol{z}_{i,\mathrm{cs}}^\top (\check{\boldsymbol{\theta}} + t\boldsymbol{\Delta})\}]^2} \mathrm{d}t \, y_i \boldsymbol{z}_{i,\mathrm{cs}}^\top \boldsymbol{\Delta} ,$$

we further deduce that

$$\begin{aligned} \lambda \check{\boldsymbol{\theta}}^\lambda &= -\int_0^1 \Bigg(n^{-1} \sum_{i=1}^n \frac{\exp\{y_i \boldsymbol{z}_{i,\mathrm{cs}}^\top (\check{\boldsymbol{\theta}} + t\boldsymbol{\Delta})\}}{[1 + \exp\{y_i \boldsymbol{z}_{i,\mathrm{cs}}^\top (\check{\boldsymbol{\theta}} + t\boldsymbol{\Delta})\}]^2} \boldsymbol{z}_{i,\mathrm{cs}} \boldsymbol{z}_{i,\mathrm{cs}}^\top \Bigg) \mathrm{d}t \, \boldsymbol{\Delta} \\ &= -\int_0^1 \Bigg(n^{-1} \sum_{i=1}^n \frac{\exp\{y_i \boldsymbol{z}_{i,\mathrm{cs}}^\top (\check{\boldsymbol{\theta}} + t\boldsymbol{\Delta})\}}{[1 + \exp\{y_i \boldsymbol{z}_{i,\mathrm{cs}}^\top (\check{\boldsymbol{\theta}} + t\boldsymbol{\Delta})\}]^2} \boldsymbol{z}_{i,\mathrm{cs}} \boldsymbol{z}_{i,\mathrm{cs}}^\top \Bigg) \mathrm{d}t \, (\check{\boldsymbol{\theta}}^\lambda - \check{\boldsymbol{\theta}}) . \end{aligned}$$

From straightforward algebra manipulations, the latter equation implies

$$\begin{aligned} \check{\boldsymbol{\theta}}^\lambda - \check{\boldsymbol{\theta}} &= -\lambda \Bigg\{ \int_0^1 \Bigg(n^{-1} \sum_{i=1}^n \frac{\exp\{y_i \boldsymbol{z}_{i,\mathrm{cs}}^\top (\check{\boldsymbol{\theta}} + t\boldsymbol{\Delta})\}}{[1 + \exp\{y_i \boldsymbol{z}_{i,\mathrm{cs}}^\top (\check{\boldsymbol{\theta}} + t\boldsymbol{\Delta})\}]^2} \boldsymbol{z}_{i,\mathrm{cs}} \boldsymbol{z}_{i,\mathrm{cs}}^\top \Bigg) \mathrm{d}t + \lambda \boldsymbol{I}_{p+1} \Bigg\}^{-1} \check{\boldsymbol{\theta}} \\ &= -\lambda \Big\{ \int_0^1 \boldsymbol{\mathcal{I}}(\check{\boldsymbol{\theta}} + t\boldsymbol{\Delta}) \mathrm{d}t + \lambda \boldsymbol{I}_{p+1} \Big\}^{-1} \check{\boldsymbol{\theta}} , \end{aligned}$$

where $\boldsymbol{\mathcal{I}}(\check{\boldsymbol{\theta}} + t\boldsymbol{\Delta})$ is the Fisher information matrix at (3) computed at parameter $\check{\boldsymbol{\theta}} + t\boldsymbol{\Delta}$.

This provides the bound

$$\|\check{\boldsymbol{\theta}}^\lambda - \check{\boldsymbol{\theta}}\|_\infty \leq \frac{(p+1)\lambda \|\check{\boldsymbol{\theta}}\|_\infty}{\lambda + \iota_{\min}\{\int_0^1 \boldsymbol{\mathcal{I}}(\check{\boldsymbol{\theta}} + t\boldsymbol{\Delta}) \mathrm{d}t\}} .$$

Since the latter inequality trivially implies $\|\boldsymbol{\Delta}\|_\infty = \|\check{\boldsymbol{\theta}}^\lambda - \check{\boldsymbol{\theta}}\|_\infty \leq (p+1)\|\check{\boldsymbol{\theta}}\|_\infty$, which further implies $\max_{1\leq i\leq n} |y_i \boldsymbol{z}_{i,\text{cs}}^\top(\check{\boldsymbol{\theta}} + t\boldsymbol{\Delta})| \leq M(p+1)\{\|\check{\boldsymbol{\theta}}\|_\infty + (p+1)\|\check{\boldsymbol{\theta}}\|_\infty\} \leq M(p+2)^2\|\check{\boldsymbol{\theta}}\|_\infty$, we deduce the bound in (S4) from the fact that $\omega(x) = e^x/(1+e^x)^2$ is even, and strictly decreasing for all $x > 0$.

From this bound, we will derive a bound for $\|\widehat{\boldsymbol{\theta}}^\lambda - \widehat{\boldsymbol{\theta}}\|_\infty$ using the relationship

$$\begin{bmatrix} \widehat{\beta}_0^\lambda - \widehat{\beta}_0 \\ \widehat{\boldsymbol{\beta}}^\lambda - \widehat{\boldsymbol{\beta}} \end{bmatrix} = \begin{bmatrix} \check{\beta}_0^\lambda - \check{\beta}_0 - \widehat{\boldsymbol{\mu}}^\top(\widehat{\boldsymbol{\beta}}^\lambda - \widehat{\boldsymbol{\beta}}) \\ \widehat{\boldsymbol{\Sigma}}^{-1}(\check{\boldsymbol{\beta}}^\lambda - \check{\boldsymbol{\beta}}) \end{bmatrix} = \begin{bmatrix} \check{\beta}_0^\lambda - \check{\beta}_0 - \widehat{\boldsymbol{\mu}}^\top \widehat{\boldsymbol{\Sigma}}^{-1}(\check{\boldsymbol{\beta}}^\lambda - \check{\boldsymbol{\beta}}) \\ \widehat{\boldsymbol{\Sigma}}^{-1}(\check{\boldsymbol{\beta}}^\lambda - \check{\boldsymbol{\beta}}) \end{bmatrix}.$$

From the above relationship, it can readily be deduced that

$$\|\widehat{\boldsymbol{\theta}}^\lambda - \widehat{\boldsymbol{\theta}}\|_\infty \leq \left\{1 + \frac{p\,\|\widehat{\boldsymbol{\mu}}\|_\infty}{\iota_{\min}(\widehat{\boldsymbol{\Sigma}})}\right\}\|\check{\boldsymbol{\theta}}^\lambda - \check{\boldsymbol{\theta}}\|_\infty .$$

Using (S4), this directly implies that

$$\begin{aligned} \|\widehat{\boldsymbol{\theta}}^\lambda - \widehat{\boldsymbol{\theta}}\|_\infty &\leq \frac{\left\{1 + \frac{p\|\widehat{\boldsymbol{\mu}}\|_\infty}{\iota_{\min}(\widehat{\boldsymbol{\Sigma}})}\right\}(p+1)\lambda\|\check{\boldsymbol{\theta}}\|_\infty}{\lambda + \omega\{(p+2)^2\|\check{\boldsymbol{\theta}}\|_\infty\}\iota_{\min}(n^{-1}\sum_{i=1}^n \boldsymbol{z}_{i,\text{cs}}\boldsymbol{z}_{i,\text{cs}}^\top)} \\ &\leq \frac{\left\{1 + \frac{p\|\widehat{\boldsymbol{\mu}}\|_\infty}{\iota_{\min}(\widehat{\boldsymbol{\Sigma}})}\right\}^2(p+1)\lambda\|\widehat{\boldsymbol{\theta}}\|_\infty}{\lambda + \omega\left[(p+2)^2\left\{1 + \frac{p\|\widehat{\boldsymbol{\mu}}\|_\infty}{\iota_{\min}(\widehat{\boldsymbol{\Sigma}})}\right\}\|\widehat{\boldsymbol{\theta}}\|_\infty\right]\iota_{\min}\left(n^{-1}\sum_{i=1}^n \boldsymbol{z}_{i,\text{cs}}\boldsymbol{z}_{i,\text{cs}}^\top\right)} . \end{aligned}$$

Under the assumption that the $\boldsymbol{x}_i$'s have finite marginal means and variances, and that their variance–covariance matrix is invertible, together with the condition $\widehat{\boldsymbol{\theta}}$ have finite components, the above expression ensures that taking λ sufficiently small yields the equivalence– up to a negligible remainder–between $\widehat{\boldsymbol{\theta}}^\lambda$ and $\widehat{\boldsymbol{\theta}}$.

Standard errors

The standard deviation of $\widehat{\beta}_j$, given by $\sqrt{\text{Var}(\widehat{\beta}_j)} = \sqrt{n^{-1}[(\mathbb{E}\{\boldsymbol{\mathcal{I}}(\beta_{0\star}, \boldsymbol{\beta}_\star)\})^{-1}]_{jj}}$ is commonly estimated using the following quantity:

$$\widehat{\sigma}_j := \frac{1}{\sqrt{n}}\left(\left[\left(\boldsymbol{\mathcal{I}}(\widehat{\beta}_0, \widehat{\boldsymbol{\beta}})\right)^{-1}\right]_{jj}\right)^{1/2} .$$

Based on the previous subsection, which implies that $\max(|\widehat{\beta}_0^\lambda - \widehat{\beta}_0|, \|\widehat{\boldsymbol{\beta}}^\lambda - \widehat{\boldsymbol{\beta}}\|_\infty)$ can be made arbitrarily small by choosing λ small, the proof of the consistency of our standard error computation procedure follows from the derivations provided in the following section, which show that $[(\boldsymbol{\mathcal{I}}(\widehat{\beta}_0^\lambda, \widehat{\boldsymbol{\beta}}^\lambda))^{-1}]_{jj} = [(\boldsymbol{\mathcal{I}}^\lambda)^{-1}]_{jj}/s_{n,j}^2$, with $\boldsymbol{\mathcal{I}}^\lambda$ defined at the beginning of Methods - *Methodology for computing standard errors of parameter estimates in VALORIS* (in main manuscript).

Computation of standard errors

Recall from above the definition of $(\check{\beta}_0^\lambda, \check{\boldsymbol{\beta}}^\lambda)$.

For $j \in \{1, \ldots, p\}$, one obtains from the relationship between $(\widehat{\beta}_0^\lambda, \widehat{\boldsymbol{\beta}}^\lambda)$ and $(\check{\beta}_0^\lambda, \check{\boldsymbol{\beta}}^\lambda)$ that

$$[\{\boldsymbol{\mathcal{I}}(\widehat{\beta}_0^\lambda, \widehat{\boldsymbol{\beta}}^\lambda)\}^{-1}]_{jj} = [\{-\nabla^2_{\beta_0,\boldsymbol{\beta}}\check{\ell}_n(\check{\beta}_0^\lambda, \check{\boldsymbol{\beta}}^\lambda)\}^{-1}]_{jj}/s_{n,j}^2 ,$$

where

$$-\nabla^2_{\beta_0,\boldsymbol{\beta}}\check{\ell}_n(\check{\beta}_0^\lambda, \check{\boldsymbol{\beta}}^\lambda) = \frac{1}{n}\sum_{i=1}^n \frac{\exp\{y_i(\check{\beta}_0^\lambda + \boldsymbol{x}_{i,\mathrm{cs}}^\top \check{\boldsymbol{\beta}}^\lambda)\}}{[1+\exp\{y_i(\check{\beta}_0^\lambda + \boldsymbol{x}_{i,\mathrm{cs}}^\top \check{\boldsymbol{\beta}}^\lambda)\}]^2}\begin{bmatrix} 1 & \boldsymbol{x}_{i,\mathrm{cs}}^\top \\ \boldsymbol{x}_{i,\mathrm{cs}} & \boldsymbol{x}_{i,\mathrm{cs}}\boldsymbol{x}_{i,\mathrm{cs}}^\top \end{bmatrix}$$

$$= \frac{1}{n}\sum_{i=1}^n \frac{\exp\{y_i(\widehat{\beta}_0^\lambda + \boldsymbol{x}_i^\top \widehat{\boldsymbol{\beta}}^\lambda)\}}{[1+\exp\{y_i(\widehat{\beta}_0^\lambda + \boldsymbol{x}_i^\top \widehat{\boldsymbol{\beta}}^\lambda)\}]^2}\begin{bmatrix} 1 & \boldsymbol{x}_{i,\mathrm{cs}}^\top \\ \boldsymbol{x}_{i,\mathrm{cs}} & \boldsymbol{x}_{i,\mathrm{cs}}\boldsymbol{x}_{i,\mathrm{cs}}^\top \end{bmatrix}.$$

Now, recall that, for each $k \in \{1,\dots,K\}$, the vector $\widehat{\boldsymbol{c}}^{\lambda(k)}$ defined in (7) satisfies $\widehat{\boldsymbol{c}}^{\lambda(k)} = \boldsymbol{X}_{\mathrm{cs}}^{(k)}\,\mathrm{diag}(s_{n,1}^{(k)},\dots,s_{n,p^{(k)}}^{(k)})\widehat{\boldsymbol{\beta}}^{\lambda(k)} = \boldsymbol{X}^{(k)}\widehat{\boldsymbol{\beta}}^{\lambda(k)} - (\sum_{j=1}^{p^{(k)}}\widehat{\beta}_j^{\lambda(k)}\mu_j^{(k)})\mathbf{1}_n$, and that the response-node has access to $\widehat{\boldsymbol{c}}^{\lambda(1)},\dots,\widehat{\boldsymbol{c}}^{\lambda(K)}$. Since the response-node can also compute $(n\lambda)^{-1}\sum_{i=1}^n \widehat{\alpha}_i^\lambda y_i = \widehat{\beta}_0^\lambda + \sum_{j=1}^n \widehat{\beta}_j^\lambda \mu_{n,j}$ (recall the expression given in (6)), it is therefore able to compute

$$\widehat{\beta}_0^\lambda\mathbf{1}_n + \boldsymbol{X}\widehat{\boldsymbol{\beta}}^\lambda = \widehat{\beta}_0^\lambda\mathbf{1}_n + \sum_{k=1}^K \boldsymbol{X}^{(k)}\widehat{\boldsymbol{\beta}}^{\lambda(k)} = ((n\lambda)^{-1}\sum_{i=1}^n \widehat{\alpha}_i^\lambda y_i)\mathbf{1}_n + \sum_{k=1}^K \widehat{\boldsymbol{c}}^{\lambda(k)}.$$

Then, upon defining $\widehat{\boldsymbol{V}}^\lambda$, whose diagonal entries $[\widehat{\boldsymbol{V}}^\lambda]_{jj}$ satisfy

$$[\widehat{\boldsymbol{V}}^\lambda]_{jj} = \frac{\exp\left[y_j\left\{(n\lambda)^{-1}\sum_{i=1}^n \widehat{\alpha}_i^\lambda y_i + \sum_{k=1}^K \widehat{c}_j^{\lambda(k)}\right\}\right]}{\left(1+\exp\left[y_j\left\{(n\lambda)^{-1}\sum_{i=1}^n \widehat{\alpha}_i^\lambda y_i + \sum_{k=1}^K \widehat{c}_j^{\lambda(k)}\right\}\right]\right)^2}$$

$$= \frac{\exp\{y_j(\widehat{\beta}_0^\lambda + \boldsymbol{x}_j^\top\widehat{\boldsymbol{\beta}}^\lambda)\}}{[1+\exp\{y_j(\widehat{\beta}_0^\lambda + \boldsymbol{x}_j^\top\widehat{\boldsymbol{\beta}}^\lambda)\}]^2},$$

the matrix $-\nabla^2_{\beta_0,\boldsymbol{\beta}}\check{\ell}_n(\check{\beta}_0^\lambda, \check{\boldsymbol{\beta}}^\lambda)$ can be computed as

$$\boldsymbol{\mathcal{I}}^\lambda = n^{-1}\begin{bmatrix}\mathbf{1}_n & \boldsymbol{X}_{\mathrm{cs}}^{(1)} & \dots & \boldsymbol{X}_{\mathrm{cs}}^{(k)}\end{bmatrix}^\top \widehat{\boldsymbol{V}}^\lambda \begin{bmatrix}\mathbf{1}_n & \boldsymbol{X}_{\mathrm{cs}}^{(1)} & \dots & \boldsymbol{X}_{\mathrm{cs}}^{(k)}\end{bmatrix}$$

$$= n^{-1}\begin{bmatrix} \mathbf{1}_n^\top\widehat{\boldsymbol{V}}^\lambda\mathbf{1}_n & \mathbf{1}_n^\top\widehat{\boldsymbol{V}}^\lambda\boldsymbol{X}_{\mathrm{cs}}^{(1)} & \cdots & \mathbf{1}_n^\top\widehat{\boldsymbol{V}}^\lambda\boldsymbol{X}_{\mathrm{cs}}^{(K)} \\ (\boldsymbol{X}^{(1)})^\top\widehat{\boldsymbol{V}}^\lambda\mathbf{1}_n & (\boldsymbol{X}_{\mathrm{cs}}^{(1)})^\top\widehat{\boldsymbol{V}}^\lambda\boldsymbol{X}_{\mathrm{cs}}^{(1)} & \cdots & (\boldsymbol{X}_{\mathrm{cs}}^{(1)})^\top\widehat{\boldsymbol{V}}^\lambda\boldsymbol{X}_{\mathrm{cs}}^{(K)} \\ \vdots & \vdots & \ddots & \vdots \\ (\boldsymbol{X}_{\mathrm{cs}}^{(K)})^\top\widehat{\boldsymbol{V}}^\lambda\mathbf{1}_n & (\boldsymbol{X}_{\mathrm{cs}}^{(K)})^\top\widehat{\boldsymbol{V}}^\lambda\boldsymbol{X}_{\mathrm{cs}}^{(1)} & \cdots & (\boldsymbol{X}_{\mathrm{cs}}^{(K)})^\top\widehat{\boldsymbol{V}}^\lambda\boldsymbol{X}_{\mathrm{cs}}^{(K)} \end{bmatrix}.$$

Supplementary Methods - Box-constrained optimization algorithm and stopping criteria

Two-metric projected Newton algorithm

The convexity of the dual problem to solve at the response-node ensures that a unique solution exists on the domain of the objective function. The algorithm used to solve the problem should allow sufficient descent to reach an adequate approximation of this unique solution. Since the components of $\boldsymbol{\alpha}$ are bounded by a compact set included in the open set $(0,1)$ (see previous Supplementary Methods), an algorithm adequate for box-constrained convex optimization problems had to be selected. While many methods exist for box-constrained optimization[5], the chosen method should allow to reach convergence with sufficient precision given the potentially small magnitude of the dual parameter $\boldsymbol{\alpha}$ while still offering efficient computation when the dimension of the dual is high. We used the Two-Metric Projected Newton method suggested by Bertsekas[6], applicable because we previously argued that the dual parameter estimates lie in a compact parameter space.

We refer to existing work from Schmidt et al.[7] for an extensive description of the method and convergence details. Briefly, all components of the estimate $\widehat{\boldsymbol{\alpha}}^{\lambda}_{(t)}$ at step t at a boundary of the search domain and for which the gradient would pull the search toward the opposite side of the search domain are updated through gradient descent projected in the domain, while all other components are updated using Newton descent projected in the domain. The update is therefore $\widehat{\boldsymbol{\alpha}}^{\lambda}_{(t+1)} = \text{Proj}[\widehat{\boldsymbol{\alpha}}^{\lambda}_{(t)} - \theta \boldsymbol{H}_{(t)} \nabla_{\boldsymbol{\alpha}} J^{\lambda}(\widehat{\boldsymbol{\alpha}}^{\lambda}_{(t)})]$, where $\boldsymbol{H}_{(t)}$ depends of the component as described before and $\text{Proj}[\cdot]$ denotes the projection under the Euclidean norm. The step size θ is selected through backtracking line search (Armijo rule) along projection arc[5,7]. An initial admissible estimate has to be provided, which was set at $\widehat{\boldsymbol{\alpha}}^{\lambda}_{(0)} = [0.1, \ldots, 0.1]^{\top}$.

Stopping criteria

The error entailed by the approximation of $\widehat{\boldsymbol{\alpha}}^{\lambda}$ in the chosen algorithm should ideally be low enough such that it preserves the asymptotic properties derived for the primal estimate. We notice that λ holds a scaling role over the dual parameter $\boldsymbol{\alpha}$ when it comes to retrieving the associated primal parameter $\boldsymbol{\beta}$. A restriction in function of λ consequently needs to be imposed in the estimation of the dual parameter to preserve the asymptotic properties of the primal parameters. We derived a stopping criteria for the dual estimation that ensures the asymptotic properties of the primal parameter hold.

We first fix $\epsilon > 0$ and consider $\tilde{\boldsymbol{\alpha}}^{\lambda} := \tilde{\boldsymbol{\alpha}}^{\lambda}_{\epsilon} \in (0,1)^n$ such that

$$\|\nabla_{\boldsymbol{\alpha}} J^{\lambda}(\tilde{\boldsymbol{\alpha}}^{\lambda})\|_2 \leq \frac{2\lambda}{\sqrt{p+1}} \Big(p(n-1)+n\Big)^{-1/2} \epsilon \,.$$

For $i \in \{1, \ldots, n\}$ let $x_{0i} = 1$ and $\breve{\beta}^{\lambda}_j = (\lambda n)^{-1} \sum_{i=1}^n y_i \tilde{\alpha}^{\lambda}_i x_{ij,\text{cs}}$ and $\breve{\beta}^{\lambda}_j = (\lambda n)^{-1} \sum_{i=1}^n y_i \widehat{\alpha}^{\lambda}_i x_{ij,\text{cs}}$ for $j \in \{0, \ldots, p\}$.

Using the fact that $y_i \in \{-1, 1\}$ for all $i \in \{1, \ldots, n\}$, we derive that

$$\begin{aligned}
\max_{j\in\{0,\ldots,p\}} |\breve{\beta}^{\lambda}_j - \breve{\beta}^{\lambda}_j| &= \max_{j\in\{0,\ldots,p\}} \Big|(\lambda n)^{-1} \sum_{i=1}^n x_{ij,\text{cs}} y_i (\tilde{\alpha}^{\lambda}_i - \widehat{\alpha}^{\lambda}_i)\Big| \\
&\leq (\lambda n)^{-1} \sum_{j=0}^p \sum_{i=1}^n |x_{ij,\text{cs}}| |\tilde{\alpha}^{\lambda}_i - \widehat{\alpha}^{\lambda}_i| \\
&\leq (\lambda n)^{-1} \|\tilde{\boldsymbol{\alpha}}^{\lambda} - \widehat{\boldsymbol{\alpha}}^{\lambda}\|_2 \sum_{j=0}^p \Big(\sum_{i=1}^n x^2_{ij,\text{cs}}\Big)^{1/2} \\
&\leq \sqrt{p+1} (\lambda n)^{-1} \|\tilde{\boldsymbol{\alpha}}^{\lambda} - \widehat{\boldsymbol{\alpha}}^{\lambda}\|_2 \Big(\sum_{j=0}^p \sum_{i=1}^n x^2_{ij,\text{cs}}\Big)^{1/2} \\
&= \sqrt{p+1} (\lambda n)^{-1} \|\tilde{\boldsymbol{\alpha}}^{\lambda} - \widehat{\boldsymbol{\alpha}}^{\lambda}\|_2 \Big(p(n-1)+n\Big)^{1/2} \,.
\end{aligned} \tag{S5}$$

To obtain the second-to-last line, we used Cauchy-Schwartz inequality, and to obtain the one-to-last line, we used the fact that for any positive $a_0, \ldots, a_p$ we have $\sum_{j=0}^p a_j \leq \sqrt{p+1} (\sum_{j=0}^p a_j^2)^{1/2}$. Finally, the last line is derived from the fact that $\sum_{i=1}^n x^2_{ij,\text{cs}} = n - 1$ for all $j \in \{1, \ldots, p\}$.

Now observe that, using standard vector calculus manipulations, the Hessian matrix of $J^{\lambda}(\boldsymbol{\alpha})$

can be expressed as

$$\nabla^2_{\boldsymbol{\alpha}} J^\lambda(\boldsymbol{\alpha}) = (\lambda n^2)^{-1}\,\mathrm{diag}(\boldsymbol{y})\Big(\sum_{k=1}^{K}\boldsymbol{\mathcal{K}}^{(k)} + \mathbf{1}_n\mathbf{1}_n^\top\Big)\,\mathrm{diag}(\boldsymbol{y}) \\ + n^{-1}\,\mathrm{diag}\left\{\left[(\alpha_1(1-\alpha_1))^{-1},\cdots,(\alpha_n(1-\alpha_n))^{-1}\right]^\top\right\}.$$

In the equation above, the matrix in the first term of the right-hand side of the equality is semi-positive definite, since for any vector $\boldsymbol{\alpha}\in\mathbb{R}^n$, $\boldsymbol{\alpha}^\top\,\mathrm{diag}(\boldsymbol{y})\Big(\sum_{k=1}^{K}\boldsymbol{\mathcal{K}}^{(k)} + \mathbf{1}_n\mathbf{1}_n^\top\Big)\,\mathrm{diag}(\boldsymbol{y})\boldsymbol{\alpha}\geq 0$. As the matrix $n^{-1}\,\mathrm{diag}\{[(\alpha_1(1-\alpha_1))^{-1},\cdots,(\alpha_n(1-\alpha_n))^{-1}]^\top\}$ is positive definite for all $\boldsymbol{\alpha}\in(0,1)^n$, with $(\alpha_i(1-\alpha_i))^{-1}\geq 4$ for all $i\in\{1,\dots,n\}$, it follows that $\nabla^2_{\boldsymbol{\alpha}}J^\lambda(\boldsymbol{\alpha})$ is strongly convex, with strong convexity parameter $m=4n^{-1}$, since it follows from the last discussion that the matrix

$$\nabla^2_{\boldsymbol{\alpha}}J^\lambda(\boldsymbol{\alpha}) - \frac{m}{2}\boldsymbol{I}$$

is positive definite.

This allows us to conclude (see e.g. Boyd et al.[8], p.459) that it holds for all $\boldsymbol{\alpha}\in(0,1)^n$ that

$$||\boldsymbol{\alpha}-\widehat{\boldsymbol{\alpha}}^\lambda||_2 \leq \frac{2}{m}\|\nabla_{\boldsymbol{\alpha}}J^\lambda(\boldsymbol{\alpha})\|_2 = \frac{n}{2}\|\nabla_{\boldsymbol{\alpha}}J^\lambda(\boldsymbol{\alpha})\|_2.$$

Combining this result with the inequality derived at (S5), we obtain

$$\max_{j\in\{0,\dots,p\}}|\breve{\beta}_j^\lambda - \check{\beta}_j^\lambda| \leq \frac{\sqrt{p+1}}{2\lambda}\|\nabla_{\boldsymbol{\alpha}}J^\lambda(\tilde{\boldsymbol{\alpha}}^\lambda)\|_2\Big(p(n-1)+n\Big)^{1/2}.$$

Now combining this result with the fact that

$$\|\nabla_{\boldsymbol{\alpha}}J^\lambda(\tilde{\boldsymbol{\alpha}}^\lambda)\|_2 \leq \frac{2\lambda}{\sqrt{p+1}}\Big(p(n-1)+n\Big)^{-1/2}\epsilon,$$

it directly follows that $\max_{j\in\{0,\dots,p\}}|\breve{\beta}_j^\lambda - \check{\beta}_j^\lambda| < \epsilon$.

It remains that we wish to establish the bound over $\max_{j\in\{0,\dots,p\}}|\tilde{\beta}_j^\lambda - \widehat{\beta}_j^\lambda|$, where $(\tilde{\beta}_0^\lambda,\tilde{\boldsymbol{\beta}}^\lambda)$ denotes a version of $(\widehat{\beta}_0^\lambda,\widehat{\boldsymbol{\beta}}^\lambda)$ computed based on $\tilde{\boldsymbol{\alpha}}^\lambda$ instead of $\widehat{\boldsymbol{\alpha}}^\lambda$. As shown in previous sections, the maximizer $(\widehat{\beta}_0^\lambda,\widehat{\boldsymbol{\beta}}^\lambda)$ of $l_n^\lambda(\beta_0,\boldsymbol{\beta})$ satisfies

$$\begin{bmatrix}\widehat{\beta}_0^\lambda\\ \widehat{\boldsymbol{\beta}}^\lambda\end{bmatrix} = \begin{bmatrix}\breve{\beta}_0^\lambda - \widehat{\boldsymbol{\mu}}^\top\widehat{\boldsymbol{\beta}}^\lambda\\ \widehat{\boldsymbol{\Sigma}}^{-1}\breve{\boldsymbol{\beta}}^\lambda\end{bmatrix},$$

where $\widehat{\boldsymbol{\mu}}$ and $\widehat{\boldsymbol{\Sigma}}$ are defined in (S1).

Therefore, $(\tilde{\beta}_0^\lambda,\tilde{\boldsymbol{\beta}}^\lambda)$ satisfies

$$\begin{bmatrix}\tilde{\beta}_0^\lambda\\ \tilde{\boldsymbol{\beta}}^\lambda\end{bmatrix} = \begin{bmatrix}\check{\beta}_0^\lambda - \widehat{\boldsymbol{\mu}}^\top\tilde{\boldsymbol{\beta}}^\lambda\\ \widehat{\boldsymbol{\Sigma}}^{-1}\check{\boldsymbol{\beta}}^\lambda\end{bmatrix},$$

Therefore, if $\tilde{\boldsymbol{\alpha}}^\lambda$ is a point such that

$$\|\nabla_{\boldsymbol{\alpha}}J^\lambda(\tilde{\boldsymbol{\alpha}}^\lambda)\|_2 \leq \frac{2\lambda}{\sqrt{p+1}}\Big(p(n-1)+n\Big)^{-1/2}\epsilon,$$

it follows that $\max_{j\in\{0,\dots,p\}}|\breve{\beta}_j^\lambda - \check{\beta}_j^\lambda| < \epsilon$. Then, for $j\in\{1,\dots,p\}$, we have

$$|\widehat{\beta}_j^\lambda - \tilde{\beta}_j^\lambda| \leq s_{n,j}^{-1}|\breve{\beta}_j^\lambda - \check{\beta}_j^\lambda| \leq s_{n,j}^{-1}\epsilon.$$

The inequality over $\|\nabla_{\boldsymbol{\alpha}}J^\lambda(\tilde{\boldsymbol{\alpha}}^\lambda)\|_2$ can therefore be used as a stopping criteria, and the asymptotic properties are preserved under $\epsilon = o(n^{-1/2})$.

Supplementary Methods - Implementation details and computational considerations of the VALORIS algorithm

The VALORIS algorithm involves several matrix and vector operations whose dimensions depend on n. When n is large, implementations that do not fully leverage the structure of the quantities involved may hinder computational feasibility. For instance, the response-node must compute the minimizer (up to tolerance) of $J^{\lambda}(\boldsymbol{\alpha})$ in (4) (main text), with $\boldsymbol{\alpha} \in (0,1)^n$. Since the Hessian $\nabla^2_{\boldsymbol{\alpha}} J^{\lambda}(\boldsymbol{\alpha})$ is an $n \times n$ matrix, Newton-Raphson updates can become computationally onerous if implemented by explicitly forming the Hessian and solving the associated linear system without exploiting its structure. Below, we describe how our implementation avoids this computational burden and outline how the computational demand scales with the sample size n, the total number of covariates involved p, and the total number of sites K.

Computations at the response-node

There are four computational steps that need to be undertaken at the response-node: (1) compute the minimizer (up to tolerance) of $J^{\lambda}(\boldsymbol{\alpha})$ in (4); (2) compute $\tilde{\boldsymbol{c}}^{\lambda(k)}$ for $k \in \{1, \ldots, K\}$, with $\tilde{\boldsymbol{c}}^{\lambda(k)}$ defined in Algorithm 1 of the manuscript; (3) Compute $\tilde{\boldsymbol{S}}^{-1}$, with $\tilde{\boldsymbol{S}} = n[(\tilde{\boldsymbol{V}}^{\lambda})^{-1} + n^{-1}\eta^{-1}(\sum_{k=1}^{K} \boldsymbol{\mathcal{K}}^{(k)} + \mathbf{1}_n \mathbf{1}_n^{\top})]$, also defined in Algorithm 1; (4) for $k \in \{1, ..., K\}$, generate $\boldsymbol{\mathcal{N}}^{(k)} \in \mathbb{R}^{n \times (n-p^{(k)})}$ in the null-space of $\boldsymbol{\mathcal{K}}^{(k)}$.

To carry out step 1, as described in Supplementary Methods - *Two-metric projected Newton algorithm*, our implementation relies on the two-metric projected Newton algorithm, which requires computing a Newton direction at each iteration, defined, for a given $\boldsymbol{\alpha} \in (0,1)^n$, as $d_{\boldsymbol{\alpha}} = \{\nabla^2_{\boldsymbol{\alpha}} J^{\lambda}(\boldsymbol{\alpha})\}^{-1} \nabla_{\boldsymbol{\alpha}} J^{\lambda}(\boldsymbol{\alpha})$, where

$$\nabla_{\boldsymbol{\alpha}} J^{\lambda}(\boldsymbol{\alpha}) = \frac{1}{\lambda n^2} \operatorname{diag}(\boldsymbol{y}) \Big(\sum_{k=1}^{K} \boldsymbol{\mathcal{K}}^{(k)} + \mathbf{1}_n \mathbf{1}_n^{\top} \Big) \operatorname{diag}(\boldsymbol{y}) \boldsymbol{\alpha} + \frac{1}{n} \left[\log\left(\frac{\alpha_1}{1-\alpha_1} \right), \cdots, \log\left(\frac{\alpha_n}{1-\alpha_n} \right) \right]^{\top}.$$

and

$$\nabla^2_{\boldsymbol{\alpha}} J^{\lambda}(\boldsymbol{\alpha}) = \frac{1}{\lambda n^2} \operatorname{diag}(\boldsymbol{y}) \Big(\sum_{k=1}^{K} \boldsymbol{\mathcal{K}}^{(k)} + \mathbf{1}_n \mathbf{1}_n^{\top} \Big) \operatorname{diag}(\boldsymbol{y}) + \frac{1}{n} \operatorname{diag} \left[\frac{1}{\alpha_1(1-\alpha_1)}, \cdots, \frac{1}{\alpha_n(1-\alpha_n)} \right]^{\top}.$$

To compute $d_{\boldsymbol{\alpha}}$ efficiently, our implementation exploits the fact that $n\nabla^2_{\boldsymbol{\alpha}} J^{\lambda}(\boldsymbol{\alpha}) = \boldsymbol{G} + \boldsymbol{D}_{\boldsymbol{\alpha}}^{-1}$, where $\boldsymbol{G} = (\lambda n)^{-1} \operatorname{diag}(\boldsymbol{y}) \{\sum_{k=1}^{K} \boldsymbol{\mathcal{K}}^{(k)} + \mathbf{1}_n \mathbf{1}_n^{\top}\} \operatorname{diag}(\boldsymbol{y})$ does not depend on $\boldsymbol{\alpha}$, and $\boldsymbol{D}_{\boldsymbol{\alpha}}$ is a diagonal matrix with entries $[\boldsymbol{D}_{\boldsymbol{\alpha}}]_{ii} = \alpha_i(1-\alpha_i)$. Then, for any matrix $\boldsymbol{Z}$ such that $\boldsymbol{Z}\boldsymbol{Z}^{\top} = \boldsymbol{G}$, the Woodbury identity yields

$$(\boldsymbol{G} + \boldsymbol{D}_{\boldsymbol{\alpha}}^{-1})^{-1} = \boldsymbol{D}_{\boldsymbol{\alpha}} - \boldsymbol{D}_{\boldsymbol{\alpha}} \boldsymbol{Z} \left(\boldsymbol{I}_{p+1} + \boldsymbol{Z}^{\top} \boldsymbol{D}_{\boldsymbol{\alpha}} \boldsymbol{Z} \right)^{-1} \boldsymbol{Z}^{\top} \boldsymbol{D}_{\boldsymbol{\alpha}}. \tag{S6}$$

In our implementation, such a matrix $\boldsymbol{Z}$, of dimension $n \times (p+1)$ (with $p+1$ corresponding to the rank of $\boldsymbol{G}$), is computed once at the beginning of the algorithm using the pivoted-Cholesky algorithm, with a one-time cost of order $\mathcal{O}(np^2)$. Each Newton direction can then be computed using the latter identity at a cost of order $\mathcal{O}(np^2 + p^3 + n^2 p)$ per iteration, which reduces to $\mathcal{O}(n^2 p)$ when $p < n$.

Carrying out step 2 requires computing $\tilde{\boldsymbol{c}}^{\lambda(k)}$ for $k \in \{1, \ldots, K\}$, which can be done efficiently at a total cost of order $\mathcal{O}(Kn^2)$.

To carry out step 3, computing $\tilde{\boldsymbol{S}}^{-1}$, recalling that $\tilde{\boldsymbol{V}}^{\lambda}$ is a diagonal $n \times n$ matrix, our implementation applies the identity (S6) with $\boldsymbol{G} = n^{-1}\eta^{-1}(\sum_{k=1}^{K} \boldsymbol{\mathcal{K}}^{(k)} + \mathbf{1}_n \mathbf{1}_n^{\top})$ and $\boldsymbol{D}_{\boldsymbol{\alpha}} = \tilde{\boldsymbol{V}}^{\lambda}$, where we compute $\boldsymbol{Z}$ such that $\boldsymbol{Z}\boldsymbol{Z}^{\top} = \boldsymbol{G}$ using the pivoted-Cholesky algorithm. This amounts to $\mathcal{O}(np^2)$

for the Cholesky factorization, and $\mathcal{O}(np^2 + p^3 + n^2 p)$ for computing the resulting version of the formula in (S6).

Finally, to sample from the null space of $\boldsymbol{\mathcal{K}}^{(k)}$, for $k \in \{1, \dots, K\}$, our implementation uses the fact that the null space of $\boldsymbol{\mathcal{K}}^{(k)}$ is equal to the null space of $(\boldsymbol{Z}^{(k)})^\top$, with $\boldsymbol{Z}^{(k)}$ a $n \times p^{(k)}$ full column rank matrix that satisfies $\boldsymbol{Z}^{(k)}(\boldsymbol{Z}^{(k)})^\top = \boldsymbol{\mathcal{K}}^{(k)}$, together with the fact that for any $\boldsymbol{z} \in \mathbb{R}^n$, the vector $\boldsymbol{z}_{\text{proj}} = \boldsymbol{z} - \boldsymbol{Z}^{(k)}\{(\boldsymbol{Z}^{(k)})^\top \boldsymbol{Z}^{(k)}\}(\boldsymbol{Z}^{(k)})^\top \boldsymbol{z}$ belongs to the null space of $\boldsymbol{\mathcal{K}}^{(k)}$. Specifically, for each $k \in \{1, \dots, K\}$, our implementation factors $\boldsymbol{\mathcal{K}}^{(k)} = \boldsymbol{Z}^{(k)}(\boldsymbol{Z}^{(k)})^\top$ using the pivoted-Cholesky algorithm, which amounts to $\mathcal{O}\{n(p^{(k)})^2\}$ operations, forms a vector $\boldsymbol{z}$ by performing n independent draws from a standard normal distribution, and compute its projection $\boldsymbol{z}_{\text{proj}}$, with requires $\mathcal{O}\{n(p^{(k)})^2\}$ operations. This procedure therefore requires at most $\mathcal{O}(Knp_\infty^2)$ operations, where $p_\infty = \max_{1 \le k \le K} p^{(k)}$.

Computations at the covariate-nodes

At each covariate-node $k \in \{1, \dots, K\}$, three standard computational steps are performed: (1) computation of the local Gram matrix $\boldsymbol{\mathcal{K}}^{(k)}$; (2) solution of a linear system of equations involving $p^{(k)}$ linearly independent rows of $\boldsymbol{X}_{\text{cs}}^{(k)}$; and (3) computation of standard errors using the formula given in Algorithm 1.

These steps rely on standard linear algebra operations and can be carried out at relatively modest computational cost. Computing $\boldsymbol{\mathcal{K}}^{(k)}$ requires $\mathcal{O}\{n^2 p^{(k)}\}$ operations, solving for $\boldsymbol{\beta}^{(k)}$ requires $\mathcal{O}\{(p^{(k)})^3\}$ operations, and computing standard errors requires $\mathcal{O}\{n(p^{(k)})^2 + n^2 p^{(k)}\}$ operations.

Supplementary Methods - Privacy-preserving properties

Theoretical details : When only parameter estimates and their standard errors are available to the response-node, without the intermediate quantities

Recall from Methods - *Methodology for computing standard errors of parameter estimates in VALORIS* (in main manuscript) that $\widehat{\sigma}_j^\lambda = \sqrt{n^{-1}[(\boldsymbol{\mathcal{I}}^\lambda + \eta \boldsymbol{I}_{p+1})^{-1}]_{j+1,j+1}}/s_{n,j}$, and where from Supplementary Methods - *Computation of standard errors*, $\boldsymbol{\mathcal{I}}^\lambda$ can be expressed as

$$\boldsymbol{\mathcal{I}}^\lambda = \frac{1}{n}\sum_{i=1}^n \frac{\exp\{y_i(\widehat{\beta}_0^\lambda + \boldsymbol{x}_i^\top \widehat{\boldsymbol{\beta}}^\lambda)\}}{[1 + \exp\{y_i(\widehat{\beta}_0^\lambda + \boldsymbol{x}_i^\top \widehat{\boldsymbol{\beta}}^\lambda)\}]^2} \begin{bmatrix} 1 & \boldsymbol{x}_{i,\text{cs}}^\top \\ \boldsymbol{x}_{i,\text{cs}} & \boldsymbol{x}_{i,\text{cs}}\boldsymbol{x}_{i,\text{cs}}^\top \end{bmatrix} .$$

Also recall that $(\widehat{\beta}_0^\lambda, \widehat{\boldsymbol{\beta}}^\lambda)$ solve the maximization problem defined in (5) in the manuscript, which therefore implies that $\widehat{\boldsymbol{\beta}}^\lambda$ satisfies

$$n^{-1}\sum_{i=1}^n \frac{y_i}{1 + \exp\{y_i(\widehat{\beta}_0^\lambda + \widehat{\boldsymbol{\mu}}^\top \widehat{\boldsymbol{\beta}}^\lambda + \boldsymbol{x}_{ics}^\top \widehat{\boldsymbol{\Sigma}}\widehat{\boldsymbol{\beta}}^\lambda)\}} \boldsymbol{x}_{i,\text{cs}} = \lambda \widehat{\boldsymbol{\Sigma}}\widehat{\boldsymbol{\beta}}^\lambda ,$$

where we have introduced

$$\widehat{\boldsymbol{\mu}} = [\mu_{n,1}, \dots, \mu_{n,p}]^\top , \qquad \widehat{\boldsymbol{\Sigma}} = \text{diag}([s_{n,1}, \dots, s_{n,p}]^\top) ,$$

see Supplementary Methods - *Details for the derivation of the dual optimization problem* for details.

Suppose, without loss of generality, that the response-node also holds covariate data and is labeled as covariate-node $k = 1$, and consider the setting where only two nodes participate in the analysis: the response-node (also acting as a covariate-node) and a single additional covariate-node. The extension to scenarios involving more than two covariate-nodes follows analogously.

To analyze the privacy risk entailed when the set $\{(\widehat{\beta}_j^{\lambda(2)}, \widehat{\sigma}_j^{\lambda(2)}) : j \in \{1, \ldots, p^{(2)}\}\}$ of parameter estimates and their associated standard errors—together with the response vector $\boldsymbol{y}$ and the covariate data it holds—is the only information available to the response-node, we adopt a deliberately more adverse scenario: for the purposes of this analysis only, we assume that, in addition to the $\widehat{\beta}_j^{\lambda(2)}$'s, the response-node has access to the full matrix $\boldsymbol{\mathcal{J}}^\lambda$ defined as

$$\boldsymbol{\mathcal{J}}^\lambda := \widehat{\boldsymbol{\Sigma}}^{-2}\left(\frac{1}{n}\sum_{i=1}^{n}\frac{\exp\{y_i(\widehat{\beta}_0^\lambda + \boldsymbol{x}_i^\top\widehat{\boldsymbol{\beta}}^\lambda)\}}{[1+\exp\{y_i(\widehat{\beta}_0^\lambda + \boldsymbol{x}_i^\top\widehat{\boldsymbol{\beta}}^\lambda)\}]^2}\boldsymbol{x}_{i,\text{cs}}\boldsymbol{x}_{i,\text{cs}}^\top + \eta\boldsymbol{I}_p\right),$$

rather than solely to the $\widehat{\sigma}_j^{\lambda(2)}$'s, which satisfy $\widehat{\sigma}_j^{\lambda(2)} = \sqrt{[(\boldsymbol{\mathcal{J}}^\lambda)^{-1}]_{jj}}$. This conservative assumption simplifies the mathematical derivations and yields an upper bound on the potential privacy risk.

When at least one continuous covariate is held by a covariate-node located outside the response-node, then candidate matrices for $\boldsymbol{X}_{\text{cs}}^{(2)}$ are column-centered and scaled matrix $\boldsymbol{A} = [\boldsymbol{a}_1^\top, \ldots, \boldsymbol{a}_n^\top]^\top$ satisfying

$$n^{-1}\sum_{i=1}^{n}\frac{y_i}{1+\exp[y_i\{b + (\boldsymbol{x}_{i,\text{cs}}^{(1)})^\top\widehat{\boldsymbol{\Sigma}}^{(1)}\widehat{\boldsymbol{\beta}}^{\lambda(1)} + \boldsymbol{a}_i^\top\boldsymbol{\Delta}\widehat{\boldsymbol{\beta}}^{\lambda(2)}\}]}\begin{bmatrix}\boldsymbol{x}_{i,\text{cs}}^{(1)}\\ \boldsymbol{a}_i\end{bmatrix} = \lambda\begin{bmatrix}\widehat{\boldsymbol{\Sigma}}^{(1)}\widehat{\boldsymbol{\beta}}^{\lambda(1)}\\ \boldsymbol{\Delta}\widehat{\boldsymbol{\beta}}^{\lambda(2)}\end{bmatrix}, \tag{S7}$$

and

$$n^{-1}\sum_{i=1}^{n}\frac{\exp\{b + (\boldsymbol{x}_{i,\text{cs}}^{(1)})^\top\widehat{\boldsymbol{\Sigma}}^{(1)}\widehat{\boldsymbol{\beta}}^{\lambda(1)} + \boldsymbol{a}_i^\top\boldsymbol{\Delta}\widehat{\boldsymbol{\beta}}^{\lambda(2)}\}}{[1+\exp\{b + (\boldsymbol{x}_{i,\text{cs}}^{(1)})^\top\widehat{\boldsymbol{\Sigma}}^{(1)}\widehat{\boldsymbol{\beta}}^{\lambda(1)} + \boldsymbol{a}_i^\top\boldsymbol{\Delta}\widehat{\boldsymbol{\beta}}^{\lambda(2)}\}]^2}\begin{bmatrix}\boldsymbol{x}_{i,\text{cs}}^{(1)}(\boldsymbol{x}_{i,\text{cs}}^{(1)})^\top & \boldsymbol{x}_{i,\text{cs}}^{(1)}\boldsymbol{a}_i^\top\\ \boldsymbol{a}_i(\boldsymbol{x}_{i,\text{cs}}^{(1)})^\top & \boldsymbol{a}_i\boldsymbol{a}_i^\top\end{bmatrix}$$
$$= \begin{bmatrix}\widehat{\boldsymbol{\Sigma}}^{(1)} & \boldsymbol{0}\\ \boldsymbol{0} & \boldsymbol{\Delta}\end{bmatrix}^2\boldsymbol{\mathcal{J}}^\lambda - \eta\boldsymbol{I}_p, \tag{S8}$$

with $\widehat{\boldsymbol{\Sigma}}^{(1)} := \text{diag}([s_{n,1}^{(1)}, \ldots, s_{n,p^{(1)}}^{(1)}])$, where $\boldsymbol{\Delta} := \text{diag}([\delta_1, \ldots, \delta_{p^{(2)}}]^\top)$ is the vector of unknown standard deviations, and b denotes the unknown intercept.

Assume temporarily that the only unknowns in the system of equations above are those associated with the (or one of the, if multiple exist) continuous variables held by covariate-node $k = 2$, and that all other entries of $\boldsymbol{A}$ are fixed and equal to those of $\boldsymbol{X}_{\text{cs}}^{(2)}$. In this case, the system comprises $n + 2$ real-valued unknowns: the n entries of the continuous variable in $\boldsymbol{A}$, one candidate intercept b, and one candidate standard deviation corresponding to the continuous variable in $\boldsymbol{\Delta}$. On the other hand, the system imposes $p + p(p+1)/2$ equality constraints: p equations from the first-order optimality condition in (S7), and $p(p+1)/2$ from the symmetry-reduced second-order condition in in (S8) (since $\boldsymbol{\mathcal{J}}^\lambda$ is symmetric, the $p(p-1)/2$ off-diagonal constraints are not independent). In addition, two further constraints are imposed to ensure that the column of $\boldsymbol{A}$ associated with the continuous variable is centered and scaled (i.e., has mean zero and variance one). Thus, the total number of equations is $p + p(p+1)/2 + 2$, while the number of unknowns remains $n + 2$.

When the inequality $p + p(p+1)/2 < n$ holds, the number of unknowns exceeds the number of independent constraints. Since a solution lying in the interior of the feasible set exists (i.e., the configuration defined by $\boldsymbol{X}_{\text{cs}}^{(k)}$), the constraint set defines a smooth manifold of positive dimension in a neighborhood of that point. Therefore, the system admits infinitely many solutions when $p + p(p+1)/2 < n$, provided that the component of $\widehat{\boldsymbol{\beta}}^{\lambda(2)}$ associated with the continuous variable is nonzero. This argument can be repeated for each continuous covariate held by covariate-node $k = 2$, showing that each associated column admits infinitely many admissible candidate configurations.

Now assume that, in addition to a continuous covariate, $\boldsymbol{X}_{\text{cs}}^{(2)}$ also includes a centered and scaled binary covariate. Consider the case where the entries of $\boldsymbol{A}$ corresponding to this binary covariate

match those of $\boldsymbol{X}_{\text{cs}}^{(2)}$, except for two entries—one originally positive and one originally negative in $\boldsymbol{X}_{\text{cs}}^{(2)}$—whose signs are flipped to preserve the column's mean and variance (such a pair always exists under the assumption that $\boldsymbol{X}_{\text{cs}}$ is not colinear with $\mathbf{1}_n$). As above, assume that all other entries of $\boldsymbol{A}$ are equal to those of $\boldsymbol{X}_{\text{cs}}^{(2)}$, except for those in the column corresponding to the (or one of the, if multiple exist) continuous covariate. Now interpret the equations in (S7) and (S8) as defining a system in which the unknowns are the entries of $\boldsymbol{A}$ associated with the continuous covariate, its associated standard error, and the unknown intercept. Because the constraints vary smoothly with respect to the continuous covariate entries of $\boldsymbol{A}$, and provided that the vector $\boldsymbol{y}$ contains at least two entries equal to 1 and at least two equal to -1, the system admits at least one solution whenever n is larger than $p + p(p+1)/2$. This is because, as the constraints behave smoothly, small changes in the continuous values can compensate for small mismatches elsewhere, allowing the system to adjust without violating the structure required by the observed quantities.

It follows that, for any entry in a column corresponding to a binary covariate in $\boldsymbol{X}_{\text{cs}}^{(2)}$, there exists an admissible candidate matrix in which that entry takes a different value.

Privacy assessment for the response vector - Empirical criterion

We now provide details regarding the ability of covariate-node k to retrieve the response-vector y from $\widehat{\boldsymbol{c}}^{\lambda(k)}$. Recall that covariate-node k has access to $\boldsymbol{\mathcal{K}}^{(k)} = \boldsymbol{X}_{\text{cs}}^{(k)}(\boldsymbol{X}_{\text{cs}}^{(k)})^\top$ and to η, and that

$$\mathbb{S}(\widehat{\boldsymbol{c}}^{\lambda(k)}) = \{\boldsymbol{y}^\dagger \in \{-1,1\}^n : \widehat{\boldsymbol{c}}^{\lambda(k)} = (n\lambda)^{-1}\boldsymbol{\mathcal{K}}^{(k)}\,\mathrm{diag}(\boldsymbol{\alpha})\boldsymbol{y}^\dagger \text{ for some } \boldsymbol{\alpha} \in (0,1)^n\}\,.$$

Since we have assumed that at least one continuous covariate is held outside of covariate-node k, $\boldsymbol{\alpha}$ can be treated as a vector with real-valued entries. Moreoever, since we have assumed that $\boldsymbol{X}^{(k)}$ has full-column rank, as $n > p^{(k)}$, the null-space of $\boldsymbol{\mathcal{K}}^{(k)}$ has dimension $n - p^{(k)} > 0$. Letting $\boldsymbol{W}$ denote an $n \times (n - p^{(k)})$ matrix of linearly independent columns spanning the null-space of $\boldsymbol{\mathcal{K}}^{(k)}$, any solution $\boldsymbol{x}_0$ satisfying $\widehat{\boldsymbol{c}}^{\lambda(k)} = (n\lambda)^{-1}\boldsymbol{\mathcal{K}}^{(k)}\boldsymbol{x}_0$ can be expressed as $\boldsymbol{x}_0 = \mathrm{diag}(\widehat{\boldsymbol{\alpha}}^\lambda)\boldsymbol{y} + \boldsymbol{W}\boldsymbol{b}$, for $\boldsymbol{b} \in \mathbb{R}^{n-p^{(k)}}$, the solution space $\mathbb{S}(\widehat{\boldsymbol{c}}^{\lambda(k)})$ can be re-expressed as

$$\mathbb{S}(\widehat{\boldsymbol{c}}^{\lambda(k)}) = \left\{\boldsymbol{y}^\dagger \in \{-1,1\}^n : \boldsymbol{y}^\dagger = \mathrm{sign}\{\mathrm{diag}(\widehat{\boldsymbol{\alpha}}^\lambda)\boldsymbol{y} + \boldsymbol{W}\boldsymbol{b}\}, \text{ with } \mathrm{diag}(\widehat{\boldsymbol{\alpha}}^\lambda)\boldsymbol{y} + \boldsymbol{W}\boldsymbol{b} \in (-1,1)^n\right\},$$

where, in the above equation, the function $\mathrm{sign}(\cdot)$, when applied to a vector, is understood component-wise: it returns -1 for each negative entry and 1 for each positive entry. To derive this expression, we also used the fact that any $\boldsymbol{y}^\dagger \in \{-1,1\}^n$ satisfying $\widehat{\boldsymbol{c}}^{\lambda(k)} = (n\lambda)^{-1}\boldsymbol{\mathcal{K}}^{(k)}\,\mathrm{diag}(\boldsymbol{\alpha})\boldsymbol{y}^\dagger$ for some $\boldsymbol{\alpha} \in (0,1)^n$ satisfies $\boldsymbol{y}^\dagger = \mathrm{sign}(\mathrm{diag}(\boldsymbol{\alpha})\boldsymbol{y}^\dagger)$.

Using these derivations, an empirical criterion was derived to verify if, using the quantities available at the covariate-nodes, every entry of the response-node's data can be flipped while still leading to an admissible candidates for the response vector. This criterion is described in Algorithm S1 to support numerical implementation.

This criterion can be verified at the response-node for any covariate-node k not co-located at the response-node.

Algorithm S1 Empirical criterion for the privacy assessment of the response vector $\boldsymbol{y}$ at covariate-node k

---

Input: Gram matrix $\boldsymbol{\mathcal{K}}^{(k)}$ from covariate-node k, response vector $\boldsymbol{y}$ and dual numerical estimate $\tilde{\boldsymbol{\alpha}}^\lambda$.
Output: Number of entries of the vector $\boldsymbol{y}$ that could be flipped.
Procedure:

1. Generate $\boldsymbol{W}$ in the null-space of $\boldsymbol{\mathcal{K}}^{(k)}$.
2. For every $i \in \{1,\ldots,n\}$, verify if $\exists\ \boldsymbol{x}_0$ such that $\mathrm{sign}(x_{0i}) \neq \mathrm{sign}(y_i)$, where $\boldsymbol{x}_0 = \mathrm{diag}(\tilde{\boldsymbol{\alpha}}^\lambda)\boldsymbol{y} + \boldsymbol{W}\boldsymbol{b} \in (-1,1)^n$.
3. Count the number of entries y_i that satisfied the condition.

Based on numerical simulations, the empirical criterion is likely to be satisfied when the sample size is sufficiently large relative to the number of covariates at node k—for example, when $n \geq 100$ and $p^{(k)} \leq 10$.

In our implementation of the empirical criterion, which relies on an open-source R solver, we observed runtime overhead over one covariate-node of approximately 10 seconds with the CKD experiment, approximately 25 seconds with our simulated data, and approximately 21 hours with the MIMIC-IV dataset. The runtime overhead largely scales with the sample size and depends on the computational environment; in our experiments, computations were performed on a standard laptop with details provided in Supplementary Tables. The verification step involves solving a series of standard feasible point problems with linear inequality constraints whose number scales with the sample size, and for which more efficient solvers than the one used in our basic implementation are available.

Supplementary Methods - Conservative Scenario: Response-Node Retains Intermediate Quantities

In real applications, a covariate-node may not assume that the response-node has executed the algorithm as intended. By analogy with the use of the term *honest-but-curious* in the main text, this situation can be viewed as involving a *malicious* adversary[9], where the malicious behavior consists solely of not performing step 4 of the algorithm—that is, without collusion with other parties.

In the following, we consider the conservative scenario in which the response-node has access to the local Gram matrices, in addition to the other disclosed estimates. The ability of the response-node to attempt reverse-engineering then depends on whether the covariate-nodes disclose p-values, or parameter estimates together with their standard errors. To analyze this ability, let

$$\mathbb{S}(\boldsymbol{\mathcal{K}}^{(k)}) = \left\{ \boldsymbol{A} \in \mathcal{M}_{n,p^{(k)}}(\mathbb{R}) \middle| \begin{array}{c} \boldsymbol{A}\boldsymbol{A}^\top = \boldsymbol{\mathcal{K}}^{(k)},\ \boldsymbol{A}^\top \mathbf{1}_n = 0, \\ \text{and } \operatorname{diag}_{\text{vec}}(\boldsymbol{A}^\top \boldsymbol{A}) = (n-1)\mathbf{1}_{p^{(k)}} \end{array} \right\}.$$

The set $\mathbb{S}(\boldsymbol{\mathcal{K}}^{(k)})$ consists of all candidate matrices for $\boldsymbol{X}_{\text{cs}}^{(k)}$ from which node k's local Gram matrix $\boldsymbol{\mathcal{K}}^{(k)}$ could equivalently be computed. When $p^{(k)} = 1$, we obtain directly that $\mathbb{S}(\boldsymbol{\mathcal{K}}^{(k)}) = \{\boldsymbol{X}_{\text{cs}}^{(k)}, -\boldsymbol{X}_{\text{cs}}^{(k)}\}$. For $p^{(k)} \geq 2$, any sign-permutation matrix $\boldsymbol{P}_\pi^{\pm}$—that is, any matrix with exactly one nonzero entry in each row and each column, with each nonzero entry equal to either 1 or -1—can be shown to satisfy $\boldsymbol{X}_{\text{cs}}^{(k)} \boldsymbol{P}_\pi^{\pm} \in \mathbb{S}(\boldsymbol{\mathcal{K}}^{(k)})$, so that $\boldsymbol{X}_{\text{cs}}^{(k)} \boldsymbol{P}_\pi^{\pm}$ is always an admissible candidate for $\boldsymbol{X}_{\text{cs}}^{(k)}$.

Note on the separation of binary and continuous covariates

In the privacy analysis, we analyze binary and continuous covariates separately. Proceeding in this way yields to conservative privacy risk assessments, since, for instance, the local Gram matrices $\boldsymbol{\mathcal{K}}^{(k,\text{bin})} = \boldsymbol{X}^{(k,\text{bin})}(\boldsymbol{X}^{(k,\text{bin})})^\top$ and $\boldsymbol{\mathcal{K}}^{(k,\text{cont})} = \boldsymbol{X}^{(k,\text{cont})}(\boldsymbol{X}^{(k,\text{cont})})^\top$ are not transmitted separately to the response-node; instead, only their sum, $\boldsymbol{\mathcal{K}}^{(k)} = \boldsymbol{\mathcal{K}}^{(k,\text{bin})} + \boldsymbol{\mathcal{K}}^{(k,\text{cont})}$, is disclosed. We proceed in this way because it remains unclear, at this stage, under which conditions the above decomposition is unique, and thus whether there is a risk that the response-node could uniquely recover $\boldsymbol{\mathcal{K}}^{(k,\text{bin})}$ and $\boldsymbol{\mathcal{K}}^{(k,\text{cont})}$ from the aggregate matrix $\boldsymbol{\mathcal{K}}^{(k)}$.

When only the local Gram matrix and parameter estimates are available to the response-node

When, in addition to $\boldsymbol{\mathcal{K}}^{(k)}$, the response node has access to a selected subset $J \subseteq \{1, \ldots, p^{(k)}\}$ of the components of the estimated parameters $\widehat{\boldsymbol{\beta}}^{\lambda(k)}$ (which may include all components), reverse-

engineering $\boldsymbol{X}_{\text{cs}}^{(k)}$ would require the response-node to search for a matrix $\boldsymbol{A} \in \mathbb{S}(\boldsymbol{\mathcal{K}}^{(k)})$ that satisfies

$$s_{n,j}^{(k)} \widehat{\beta}_j^{\lambda(k)} = (n\lambda)^{-1} \sum_{i=1}^{n} [\boldsymbol{A}]_{ij} \widehat{\alpha}_i^{\lambda} y_i \quad \text{for all } j \in J \ ,$$

where the values $s_{n,j}^{(k)}$ are not available to the response-node.

When $\boldsymbol{X}_{\text{cs}}^{(k)}$ is computed from continuous covariate data, successful reverse-engineering of node k's data would require the response-node to identify a matrix $\boldsymbol{A}$ such that there exists a set of constants $\{a_j : j \in J\}$ with strictly positive entries ($a_j > 0$ for all j) satisfying

$$a_j \widehat{\beta}_j^{\lambda(k)} = (n\lambda)^{-1} \sum_{i=1}^{n} [\boldsymbol{A}]_{ij} \widehat{\alpha}_i^{\lambda} y_i \quad \text{for all } j \in J \ . \tag{S9}$$

Now for any $\boldsymbol{A} \in \mathbb{S}(\boldsymbol{\mathcal{K}}^{(k)})$, if $\boldsymbol{A}$ does not satisfy (S9), there exists a set of indices $J' \subseteq J$ such that $(\widehat{\beta}_j^{\lambda(k)} n\lambda)^{-1} \sum_{i=1}^{n} [\boldsymbol{A}]_{ij} \widehat{\alpha}_i^{\lambda} y_i < 0$ for all $j \in J'$. Since flipping the signs of all entries in any given column of a matrix in $\mathbb{S}(\boldsymbol{\mathcal{K}}^{(k)})$ yields another matrix that also belongs to $\mathbb{S}(\boldsymbol{\mathcal{K}}^{(k)})$, it follows that the matrix $\boldsymbol{A}' = \boldsymbol{A} \prod_{j \in J'} \boldsymbol{P}_j^{\pm}$ still belongs to $\mathbb{S}(\boldsymbol{\mathcal{K}}^{(k)})$ while now meeting the constraint given by (S9). We draw the following conclusions:

- Case $p^{(k)} = 1$: In this case, as $\mathbb{S}(\boldsymbol{\mathcal{K}}^{(k)}) = \{\boldsymbol{X}_{\text{cs}}^{(k)}, -\boldsymbol{X}_{\text{cs}}^{(k)}\}$, for any $\boldsymbol{A} \in \mathbb{S}(\boldsymbol{\mathcal{K}}^{(k)})$, only one of $\boldsymbol{A}$ and $\boldsymbol{A}' = \boldsymbol{A}\boldsymbol{P}_1^{\pm}$ will meet (S9). Centered and scaled individual-level data could be retrieved.
- Case $p^{(k)} \geq 2$: Given that matrices $\boldsymbol{A} = \boldsymbol{X}_{\text{cs}}^{(k)} \boldsymbol{P}_{\pi}^{\pm}$ are in $\mathbb{S}(\boldsymbol{\mathcal{K}}^{(k)})$, it follows that at least $\boldsymbol{A} = \boldsymbol{X}_{\text{cs}}^{(k)} \boldsymbol{P}_{\pi}$, where $\boldsymbol{P}_{\pi}$ denotes a permutation matrix (i.e., either the identity matrix, or the matrix that permutes the columns) will meet constraint (S9).

In the binary-covariates case, as the binary nature must be preserved, it can be shown that candidate matrices for $\boldsymbol{X}_{\text{cs}}^{(k)}$ are all of the form $\boldsymbol{A} = \boldsymbol{X}_{\text{cs}}^{(k)} \boldsymbol{P}_{\pi}^{\pm}$. Therefore, if the response-node is able to identify a single admissible candidate in $\mathbb{S}(\boldsymbol{\mathcal{K}}^{(k)})$, it can compute all candidates in $\mathbb{S}(\boldsymbol{\mathcal{K}}^{(k)})$. The binary nature of the covariates involved at node k implies that the entries $\boldsymbol{X}_{\text{cs}}^{(k)}$ have the form

$$[\boldsymbol{X}_{\text{cs}}^{(k)}]_{ij} = \frac{x_{ij}^{(k)} - \mu_j}{\sqrt{\mu_j(1-\mu_j)}} \sqrt{\frac{n-1}{n}} \ , \quad x_{ij}^{(k)} \in \{0,1\} \ , \quad \mu_j \in \left\{\frac{1}{n}, \ldots, \frac{n-1}{n}\right\} .$$

This implies that the proportion of positive entries in the jth column of $\boldsymbol{X}_{\text{cs}}^{(k)}$ is equal to $\mu_{n,j}^{(k)}$, and the standard deviation of the covariate in column j can therefore be computed as

$$\sqrt{\frac{n}{n-1}} \sqrt{\left\{n^{-1} \sum_{i=1}^{n} \mathbb{I}(x_{ij,\text{cs}}^{(k)} > 0)\right\}\left\{1 - n^{-1} \sum_{i=1}^{n} \mathbb{I}(x_{ij,\text{cs}}^{(k)} > 0)\right\}} \ ,$$

where $\mathbb{I}(B)$ is the indicator function taking value 1 if B is true, and 0 otherwise. Then, recalling equation (S9), if $\widehat{\beta}_j^{(k)}$ is disclosed for all $j \in J$, the response-node can narrow its search for $\boldsymbol{X}_{\text{cs}}^{(k)}$ by identifying which admissible candidate $\boldsymbol{A} \in \mathbb{S}(\boldsymbol{\mathcal{K}}^{(k)})$ satisfies the following equality:

$$\sqrt{\frac{n}{n-1}} \sqrt{\mu_j^A (1 - \mu_j^A)} = (\widehat{\beta}_j^{\lambda(k)} n\lambda)^{-1} \sum_{i=1}^{n} A_{ij} \widehat{\alpha}_i^{\lambda} y_i \quad \text{for all } j \in J \ , \tag{S10}$$

with $\mu_j^A = n^{-1}\sum_{i=1}^n \mathbb{I}([\boldsymbol{A}]_{ij} > 0)$. This typically yields a set of admissible candidates with a unique possibility for every column $j \in J$ of $\boldsymbol{X}_{\text{cs}}^{(k)}$, irrespective of the cardinality of J.

Therefore, in the conservative non-colluding malicious adversary setting, disclosing parameter estimates for binary covariates entails a risk of data leakage.

When only the local Gram matrix, parameter estimates and their standard errors are available to the response-node

We now turn to the solution space of candidate matrices $\boldsymbol{A}$ when coefficient estimates indexed by $J \subseteq \{1, \ldots, p^{(k)}\}$ are disclosed, and for a subset of these, $J_{\text{sd}} \subseteq J$, the corresponding standard errors are also released. We restrict attention to the case of continuous covariates, since, as shown previously, the disclosure of parameter estimates for binary covariates entails a risk of data leakage.

Recall the expression of $\widehat{\sigma}_j^{\lambda(k)}$ for $j \in \{1, \ldots, p^{(k)}\}$, given by

$$s_{n,j}^{(k)}\widehat{\sigma}_j^{\lambda(k)} = \left\{n^{-1}\eta^{-1} - n^{-1}\eta^{-2}[(\boldsymbol{X}_{\text{cs}}^{(k)})^\top \widehat{\boldsymbol{S}}^{-1}\boldsymbol{X}_{\text{cs}}^{(k)}]_{jj}\right\}^{1/2}. \tag{S11}$$

While the values $s_{n,j}^{(k)}$ are not known to the response-node, for $j \in J$ the coefficients are $\widehat{\beta}_j^{(k)}$. Moreover, from (S9), a candidate $\boldsymbol{A} \in \mathbb{S}(\boldsymbol{\mathcal{K}}^{(k)})$ implicitly determines a corresponding candidate value for $s_{n,j}^{(k)}$, given by $a_j = (\widehat{\beta}_j^{\lambda(k)} n\lambda)^{-1}\sum_{i=1}^n [\boldsymbol{A}]_{ij}\widehat{\alpha}_i^\lambda y_i$. Consequently, reverse-engineering $\boldsymbol{X}_{\text{cs}}^{(k)}$ requires the response-node to search for $\boldsymbol{A} \in \mathbb{S}(\boldsymbol{\mathcal{K}}^{(k)})$ that satisfies (S9) for all $j \in J$, as well as the following for all $j \in J_{\text{sd}}$:

$$n^{-1}\eta^{-1} - n^{-1}\eta^{-2}\sum_{m=1}^n\sum_{i=1}^n [\boldsymbol{A}]_{ij}[\boldsymbol{A}]_{mj}[\widehat{\boldsymbol{S}}^{-1}]_{im} = \left(\frac{\widehat{\sigma}_j^{\lambda(k)}}{\widehat{\beta}_j^{\lambda(k)} n\lambda}\sum_{i=1}^n [\boldsymbol{A}]_{ij}\widehat{\alpha}_i^\lambda y_i\right)^2. \tag{S12}$$

We next show that, when $p^{(k)} \geq 4$ and $|J_{\text{sd}}| \leq (p^{(k)}-1)(p^{(k)}-2)/2 - 1$ and $\boldsymbol{X}_{\text{cs}}^{(k)}$ has full column rank, the set of candidate matrices $\boldsymbol{A} \in \mathbb{S}(\boldsymbol{\mathcal{K}}^{(k)})$ that simultaneously satisfy (S9) for all $j \in J$ and (S12) for all $j \in J_{\text{sd}}$ is infinite.

To do so, for $\boldsymbol{\theta} \in (-\pi, \pi]^{p^{(k)}(p^{(k)}-1)/2}$, let $\boldsymbol{P_\theta}$ be defined as:

$$\boldsymbol{P_\theta} = \prod_{i=1}^{p^{(k)}-1}\prod_{j=i+1}^{p^{(k)}} \boldsymbol{\mathcal{G}}(\theta_{(i-1)(p^{(k)}-i/2)+(j-i)}; i, j)\,, \tag{S13}$$

where, for $1 \leq i < j \leq p^{(k)}$ and $\theta \in (-\pi, \pi]$, $\boldsymbol{\mathcal{G}}(\theta; i, j) \in \mathcal{M}_{p^{(k)}\times p^{(k)}}(\mathbb{R})$ denotes the Givens rotation matrix (see e.g.[10]) with entries $[\boldsymbol{\mathcal{G}}(\theta; i, j)]_{\ell\ell'}$ defined as

$$[\boldsymbol{\mathcal{G}}(\theta; i, j)]_{\ell\ell'} = \begin{cases} \cos(\theta) & \text{if } (\ell, \ell') \in \{(i,i), (j,j)\}\,, \\ -\sin(\theta) & \text{if } (\ell, \ell') = (i,j)\,, \\ \sin(\theta) & \text{if } (\ell, \ell') = (j,i)\,, \\ 1 & \text{if } (\ell, \ell') \in \{(1,1), \ldots, (p^{(k}, p^{(k)})\} \setminus \{(i,i),(j,j)\}\,, \\ 0 & \text{elsewhere}\,. \end{cases} \tag{S14}$$

In this notation, the set $\{\boldsymbol{P_\theta} : \boldsymbol{\theta} \in (-\pi, \pi]^{p^{(k)}(p^{(k)}-1)/2}\}$ is exactly the set of all rotation matrices of size $p^{(k)} \times p^{(k)}$, from which the full set of orthogonal matrices can be generated by additionally allowing reflections.

Consider $\boldsymbol{g}(\boldsymbol{\theta}) = [g_1(\boldsymbol{\theta}), \ldots, g_{p^{(k)}-1}(\boldsymbol{\theta})]$, where, for $j \in \{1, \ldots, p^{(k)} - 1\}$,

$$g_j(\boldsymbol{\theta}) = \sum_{\ell=1}^{p^{(k)}-1} \sum_{\ell'=\ell+1}^{p^{(k)}} [\boldsymbol{P}_{\boldsymbol{\theta}}]_{\ell j} [\boldsymbol{P}_{\boldsymbol{\theta}}]_{\ell' j} \tau_{\ell\ell'}^{(k)},$$

where $\tau_{\ell\ell'}^{(k)} = n^{-1} \sum_{i=1}^{n} x_{i\ell,\text{cs}}^{(k)} x_{i\ell',\text{cs}}^{(k)}$. Since $\boldsymbol{A}_{\boldsymbol{\theta}} = \boldsymbol{X}_{\text{cs}}^{(k)} \boldsymbol{P}_{\boldsymbol{\theta}}$ is centered and scaled matrix if and only if $\boldsymbol{g}(\boldsymbol{\theta}) = 0$, it can be readily verified that that

$$\left\{ \boldsymbol{A} = \boldsymbol{X}_{\text{cs}}^{(k)} \boldsymbol{P}_{\boldsymbol{\theta}} \boldsymbol{P}_{\pi}^{\pm} \mid \boldsymbol{\theta} \in (-\pi, \pi]^{p^{(k)}(p^{(k)}-1)/2} \text{ and } \boldsymbol{g}(\boldsymbol{\theta}) = \boldsymbol{0} \right\} = \mathbb{S}(\boldsymbol{\mathcal{K}}^{(k)}) \,.$$

Moreover, such $\boldsymbol{A}_{\boldsymbol{\theta}}$ satisfies (S12) for all $j \in J_{\text{sd}}$ if and only if $\boldsymbol{h}_{J_{\text{sd}}}(\boldsymbol{\theta}) = 0$, where $\boldsymbol{h}(\boldsymbol{\theta}) = [h_1(\boldsymbol{\theta}), \ldots, h_{p^{(k)}}(\boldsymbol{\theta})]$ $[h_1(\boldsymbol{\theta}), \ldots, h_{p^{(k)}}(\boldsymbol{\theta})]$ with

$$h_j(\boldsymbol{\theta}) = \frac{1}{n\eta} - \frac{1}{n\eta^2} \sum_{\ell=1}^{p^{(k)}} \sum_{\ell'=1}^{p^{(k)}} [\boldsymbol{P}_{\boldsymbol{\theta}}]_{\ell j} [\boldsymbol{P}_{\boldsymbol{\theta}}]_{\ell' j} [(\boldsymbol{X}_{\text{cs}}^{(k)})^{\top} \widehat{\boldsymbol{S}}^{-1} \boldsymbol{X}_{\text{cs}}^{(k)}]_{\ell\ell'} - \Big(\frac{\widehat{\sigma}_j^{\lambda(k)}}{\widehat{\beta}_j^{\lambda(k)}} \sum_{\ell=1}^{p^{(k)}} [\boldsymbol{P}_{\boldsymbol{\theta}}]_{\ell j} s_{n,\ell}^{(k)} \widehat{\beta}_{\ell}^{(k)} \Big)^2 .$$

Now note that the cardinality of the set $\{\boldsymbol{\theta} \in (-\pi, \pi]^{p^{(k)}(p^{(k)}-1)/2} : \boldsymbol{g}(\boldsymbol{\theta}) = \boldsymbol{0} \text{ and } \boldsymbol{h}_{J_{\text{sd}}}(\boldsymbol{\theta}) = \boldsymbol{0}\}$ is infinite. This follows directly from the smoothness of $\boldsymbol{g}(\boldsymbol{\theta}), \boldsymbol{h}_{J_{\text{sd}}}(\boldsymbol{\theta})$, the fact that if $|J_{\text{sd}}| \leq (p^{(k)} - 1)(p^{(k)} - 2)/2 - 1$ the dimension of $\boldsymbol{\theta}$ exceeds the number of equations, the existence of a solution $\boldsymbol{\theta}_0 \in (-\pi, \pi)^{p^{(k)}(p^{(k)}-1)/2}$ satisfying this solution set (i.e., the solution $\boldsymbol{\theta} = \boldsymbol{0}$, which corresponds to $\boldsymbol{P}_{\boldsymbol{\theta}} = \boldsymbol{I}_{p^{(k)}}$), and an application of the Implicit Function Theorem.

Since for any $\boldsymbol{A}_{\boldsymbol{\theta}} = \boldsymbol{X}_{\text{cs}}^{(k)} \boldsymbol{P}_{\boldsymbol{\theta}}$ with $\boldsymbol{g}(\boldsymbol{\theta}) = \boldsymbol{0}$ and $\boldsymbol{h}_{J_{\text{sd}}}(\boldsymbol{\theta}) = \boldsymbol{0}$ we have $\boldsymbol{A}_{\boldsymbol{\theta}} \in \mathbb{S}(\boldsymbol{\mathcal{K}}^{(k)})$, and as all matrices of the form $\boldsymbol{A}_{\boldsymbol{\theta}} \boldsymbol{P}^{\pm}$ satisfy both $\boldsymbol{A}_{\boldsymbol{\theta}} \boldsymbol{P}^{\pm} \in \mathbb{S}(\boldsymbol{\mathcal{K}}^{(k)})$ and (S12), the proof that the set of candidate matrices $\boldsymbol{A} \in \mathbb{S}(\boldsymbol{\mathcal{K}}^{(k)})$ that simultaneously satisfy (S9) for all $j \in J$ and (S12) for all $j \in J_{\text{sd}}$ is infinite follows from the fact that for any $\boldsymbol{\theta}$ there always exists a $\boldsymbol{P}^{\pm}$ such that $\boldsymbol{A}_{\boldsymbol{\theta}} \boldsymbol{P}^{\pm}$ satisfies (S9).

It follows that the targeted estimates and standard errors can be disclosed by the covariate-node while ensuring Privacy Level II.

When only the local Gram matrix and two-sided p-values are available to the response-node

We now study the solution space of candidate matrices $\boldsymbol{A}$ for $\boldsymbol{X}_{\text{cs}}^{(k)}$ when a subset $J \subseteq \{1, \ldots, p^{(k)}\}$ of two-sided p-values $\rho_{\text{val},j}^{\lambda(k)}$ are available to the response-node, in addition to the local Gram matrix $\boldsymbol{\mathcal{K}}^{(k)}$. Given the one-to-one relationship between $\rho_{\text{val},j}^{\lambda(k)}$ and $|\beta_j^{\lambda(k)}|/\widehat{\sigma}_j^{\lambda(k)}$, and using equations (S9) and (S11), for a candidate matrix $\boldsymbol{A}$ for $\boldsymbol{X}_{\text{cs}}^{(k)}$ to be such that the disclosed quantities $\boldsymbol{\mathcal{K}}^{(k)}$ and $\boldsymbol{\rho}_{\text{val},J}^{\lambda(k)} := \{\rho_{\text{val},j}^{\lambda(k)} : j \in J\}$ could have been equivalently computed from either $\boldsymbol{A}$ or $\boldsymbol{X}_{\text{cs}}^{(k)}$, the response-node must identify a matrix $\boldsymbol{A}$ such that

$$(\rho_{\text{val,j}}^{\lambda(k)})^2 \Big(n^{-1}\eta^{-1} - n^{-1}\eta^{-2} \sum_{m=1}^{n} \sum_{i=1}^{n} [\boldsymbol{A}]_{ij} [\boldsymbol{A}]_{mj} [\widehat{\boldsymbol{S}}^{-1}]_{im} \Big) = \Big(\frac{1}{n\lambda} \sum_{i=1}^{n} A_{ij} \widehat{\alpha}_i^{\lambda} y_i \Big)^2 . \tag{S15}$$

It can be easily verified that $\{\boldsymbol{X}_{\text{cs}}^{(k)}, -\boldsymbol{X}_{\text{cs}}^{(k)}\}$ are admissible candidates that respect equation (S15).

In Figure S1, we illustrated the conditions under which a covariate-node holding only continuous covariates can disclose p-values, parameter estimates and standard errors while achieving Privacy

Level II. The privacy-preserving properties for the case of a covariate-node k holding only continuous covariates are illustrated in Figure S1.

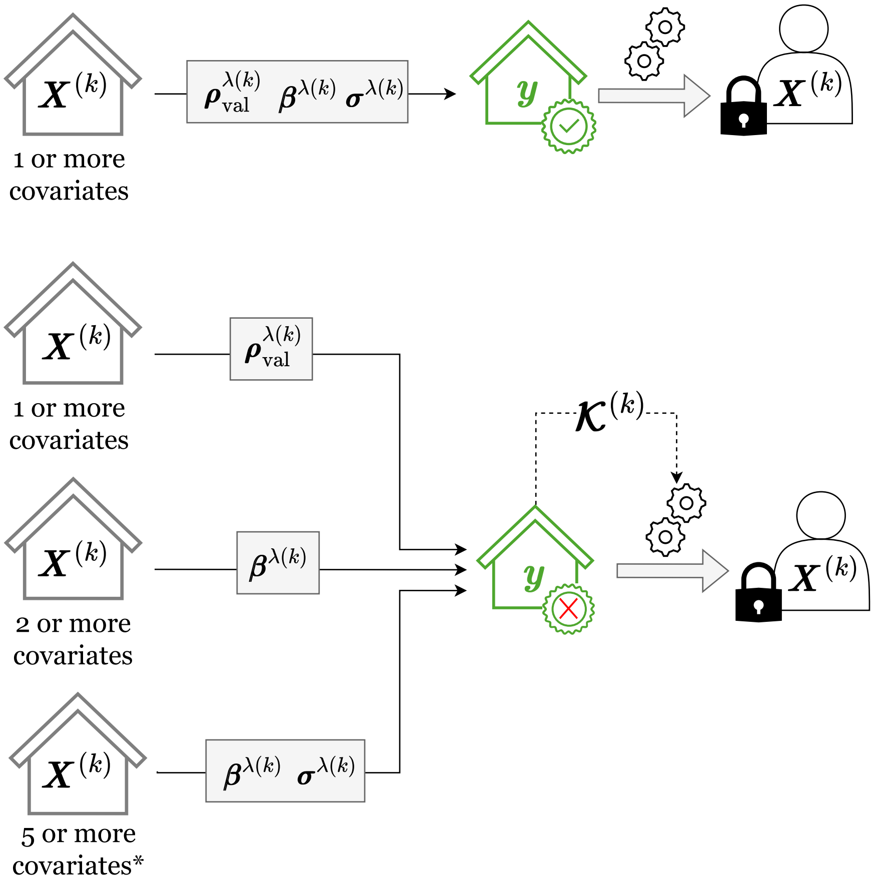

Fig. S1. Privacy-preserving properties for a covariate-node k holding continuous covariates only. The response-nodes is assumed to have cleared intermediate quantities in the upper figure, and it assumed to have not cleared intermediate quantities in the lower figure. *If $\boldsymbol{X}^{(k)}$ holds 4 continuous covariates, Privacy Level II is guaranteed when at most two pairs of estimates and standard errors are disclosed. If $\boldsymbol{X}^{(k)}$ holds 5 or more continuous covariates, Privacy Level II is guaranteed all pairs are disclosed.

References

1. Tangri, N. *et al.* A Predictive Model for Progression of Chronic Kidney Disease to Kidney Failure. *JAMA* **305**, 1553–1559, DOI: 10.1001/jama.2011.451 (2011).
2. Li, Y., Jiang, X., Wang, S., Xiong, H. & Ohno-Machado, L. VERTIcal grid lOgistic regression (VERTIGO). *J. Am. Med. Informatics Assoc.* **23**, 570–579, DOI: 10.1093/jamia/ocv146 (2016).
3. Wedderburn, R. W. M. On the Existence and Uniqueness of the Maximum Likelihood Estimates for Certain Generalized Linear Models. *Biometrika* **63**, 27–32, DOI: 10.2307/2335080 (1976).
4. Albert, A. & Anderson, J. A. On the existence of maximum likelihood estimates in logistic regression models. *Biometrika* **71**, 1–10 (1984).
5. Bertsekas, D. P. *Nonlinear Programming* (Athena Scientific, Belmont, MA, USA, 1999).

6. Bertsekas, D. P. Projected Newton Methods for Optimization Problems with Simple Constraints. *SIAM J. on Control. Optim.* **20**, 221–246, DOI: 10.1137/0320018 (1982).

7. Schmidt, M., Kim, D. & Sra, S. Projected Newton-type Methods in Machine Learning. In *Optimization for Machine Learning*, 305–330, DOI: 10.7551/mitpress/8996.003.0013 (The MIT Press, Cambridge, MA, USA, 2011).

8. Boyd, S. P. & Vandenberghe, L. *Convex optimization* (Cambridge university press, Cambridge, United Kingdom, 2004).

9. Aggarwal, C. C. & Yu, P. S. *Privacy-Preserving Data Mining: Models and Algorithms* (Springer, New York, NY, USA, 2008).

10. Givens, W. Computation of Plane Unitary Rotations Transforming a General Matrix to Triangular Form. *J. Soc. for Ind. Appl. Math.* **6**, 26–50 (1958).
